# Enhanced RF analog linearity in metal gate modulated heterojunction based uniform TFET for label-free detection of dengue NS1 protein

**DOI:** 10.1038/s41598-025-08892-5

**Published:** 2025-07-05

**Authors:** Ranjith Kumar T, Lakshmi Priya G

**Affiliations:** 1 Centre for Advanced Materials and Innovative Technologies, Vellore Institute of Technology, Chennai, 600127 India; 2 School of Electronics Engineering, Vellore Institute of Technology, Chennai, 600127 India

**Keywords:** NS1 protein sensing, Biosensor, Uniform TFET, Dielectric cavities, Gain bandwidth product, Sensitivity, Nanoscale devices, Electronic devices, Electrical and electronic engineering

## Abstract

This work presents a comprehensive investigation of symmetric (HJ-DD-UTFET) and asymmetric Source Drain Heterojunction Dual Dielectric Uniform Tunnel Field-Effect Transistors (A-SD-HJ-DD-UTFET) to achieve enhanced analog/RF, and linearity performance. The A-SD-HJ-DD-UTFET showcases an extremely low OFF current level of 8.124 × 10^–17^ A/μm which surpasses the symmetric HJ-DD-UTFET by 5,470 times and presents a high ON–OFF ratio of 2.83 × 10^12^ representing a 6,261 times improvement. This enhanced performance occurs because of structural asymmetry which makes it suitable for high-end RF and biosensing purposes while reaching a peak transconductance of 536 µS. For dengue NS1 protein detection (κ = 78.7), the TCAD-driven model of the proposed A-SD-HJ-DD-UTFET biosensor delivers a distinctive label-free detection method, achieving a peak transconductance (g_m_) of 577 µS, cut-off frequency (f_T_) of 193 GHz, Gain-Bandwidth Product (GBP) of 201 GHz, Transconductance Generation factor (TGF) of 155 V^-1^, and gain transconductance frequency product (GTFP) of 25.9 THz. These correspond to improvements of 51.4%, 13.5%, 26.4%, 96.4%, and 45.5%, respectively, over SARS-CoV spike protein detection (κ = 2). The A-SD-HJ-DD-UTFET biosensor also exhibits superior linearity performance during dengue NS1 protein detection through its desirable intercept points, minimal intermodulation distortion, and a well-maintained 1 dB compression point, affirming its potential as a high-speed, label-free RF biosensor for infectious disease Point of Care Testing (POCT) diagnostics.

## Introduction

The continuous evolution of semiconductor technology, driven by Moore’s Law, has brought about unprecedented advancements in device scaling and performance^1,2^. As traditional MOSFET architectures face challenges like Short Channel Effects (SCEs), increased leakage current, and Subthreshold Swing (SS) limitations, researchers have turned to alternative devices for enhanced energy efficiency, RF performance, and sensing capabilities^3–6^. Tunnel Field-Effect Transistors (TFETs) have emerged as a future-ready alternatives due to their unique Band-to-Band tunneling (BTBT) mechanism, enabling sub-60 mV/dec Subthreshold Swing, low OFF-state current, and CMOS compatibility^7–10^. Conventional TFETs, employing abrupt p-n junctions for tunneling, exhibit steep SS but suffer from low ON state current and fabrication complexity due to precise doping requirements. Among TFET variants, the Junctionless TFET (JLTFET) has garnered significant attention, particularly for RF and biosensing applications, owing to its simpler fabrication process and immunity to random dopant fluctuations. Although various TFET designs, including gate-engineered^11–14^, heterostructure-based^15–17^, high-k dielectric-integrated^18^, dopingless^19–21^, and vertical TFETs^22,23^, as well as cutting edge nanosheet^24–27^, nanowire^28–30^, nanotube architectures^31^ and negative capacitance-based devices^25,32,33^ have shown significant promise in sub-5 nm and analog/RF/mixed-signal domains, the proposed UTFET biosensor stands out by offering fabrication simplicity, improved linearity, and serotype-independent biosensing sensitivity without relying on ferroelectric stacks, spacer engineering, or charge plasma complexities.

JLTFETs demonstrate fabrication simplicity and reduced variability while maintaining enhanced device performance. Junctionless TFETs (JLTFETs), using uniformly doped source, channel, and drain regions, simplify fabrication while maintaining steep SS and low OFF state current^34–37^. Several JLTFET variants have been developed to overcome the inherent limitation of low ON-state current caused by tunneling barriers. Double Gate JLTFETs (DG-JLTFETs), including structures with segmented gates such as Tunnel Gate (TG) and Auxiliary Gate (AG) as Control Gate (CG) offer improved electrostatic control and wider tunneling regions, enhancing RF and subthreshold performance^38^. Gate material engineering is further explored in Dual Material Gate (DMG) and Dual Material Control Gate (DMCG) JLTFETs, which optimize tunneling and suppress ambipolarity through tailored work functions and doping profiles^39,40^.

Heterostructure JLTFETs (HJLTFETs) leverage materials like Ge, InAs/AlGaSb, and SiGe/GaAs at the source-channel interface to enhance band-to-band tunneling (BTBT) and reduce leakage, with designs such as p⁺ pocket and stacked gate structures showing strong analog/RF potential^41–44^. Charge Plasma JLTFETs eliminate traditional junctions using work function-engineered gates to induce P-I-N profiles in intrinsic silicon, improving scalability and gate control^45^.

To further boost performance, bandgap and oxide engineering approaches have been adopted. These include the use of dual-oxide layers (SiO_2_/HfO_2_), high-κ materials, and hetero material gates (HMG/SMG), all contributing to better gate-channel coupling and RF/linearity metrics^46–48^. Additionally, Negative Capacitance JLTFETs (NC-JLTFETs) using ferroelectric dielectrics like Si-doped HfO_2_, and Vertical JLTFETs (V-JLTFETs) employing stacked SiGe and HfO_2_, provide power-efficient switching and reduced device footprint while retaining analog/RF integrity^49,50^.

While advances in TFETs have improved performance, conventional JLTFETs still face limitations in RF response and linearity, particularly in biosensing applications requiring high-speed and low-power operation. Traditional SiNW FET biosensors operate at low frequencies and are prone to noise, limiting accuracy for detecting biomolecules like viral proteins^51^. Though dielectric modulation has been explored to reduce noise^52–54^ and high-κ dielectrics integrated into JLTFETs for RF enhancement^55,56^, symmetric designs still suffer from low ON-state current, poor OFF-state control, and high SS due to uniform electrostatics. While dopingless JLTFETs use charge plasma or gate work-function engineering to avoid physical junctions, the proposed UTFET employs uniform doping across source, channel, and drain^53,57,58^. Unlike JLTFETs, it simplifies fabrication by eliminating doping gradients or charge plasma layers, and introduces asymmetry and heterojunctions to boost tunneling efficiency, electrostatics, and RF performance.

To further enhance RF operation, the proposed biosensor integrates a Si–GaAs–Si heterostructure along with dual high-κ dielectrics, both feasible via advanced fabrication^59–62^. This combination lowers tunneling barriers, improves carrier mobility, and supports operation at higher frequencies—a novel configuration not previously reported for RF biosensing. The Asymmetric Source-Drain Heterojunction Dual-Dielectric Uniform TFET (A-SD-HJ-DD-UTFET) addresses key limitations by enhancing source tunneling and suppressing ambipolar leakage at the drain, improving ON-state current and ON/OFF ratio^63^. Optimized RF parameters such as Gain Bandwidth Product and transit time make it suitable for high-frequency, linear biosensing. This study targets dengue NS1 protein detection, a key biomarker secreted early in infection, enabling prompt diagnosis^64–67^. The device leverages high-frequency operation to suppress flicker noise, delivering faster response, higher SNR, and superior accuracy. Dielectric constant modulation due to NS1 interaction directly impacts the device characteristics, enabling precise, real-time biosensing.

By addressing the bottlenecks in symmetric designs and demonstrating improved RF performance for dengue NS1 detection, the A-SD-HJ-DD-UTFET offers a transformative solution for reliable, high-speed biosensors that meet emerging diagnostic demands. The device structure and operation are given in the Section “Device structure and operation”, Models used and simulation setup in Section “Models used and simulation setup” followed by RF/analog performance and linearity performance of the device in Sections “Analog/RF figure of merits” and “Linearity analysis” respectively and the performance of the A-SD-HJ-DD-UTFET biosensor in Section “RF, analog and linearity performance of biosensor”.

## Device structure and operation

**Fig. 1:**
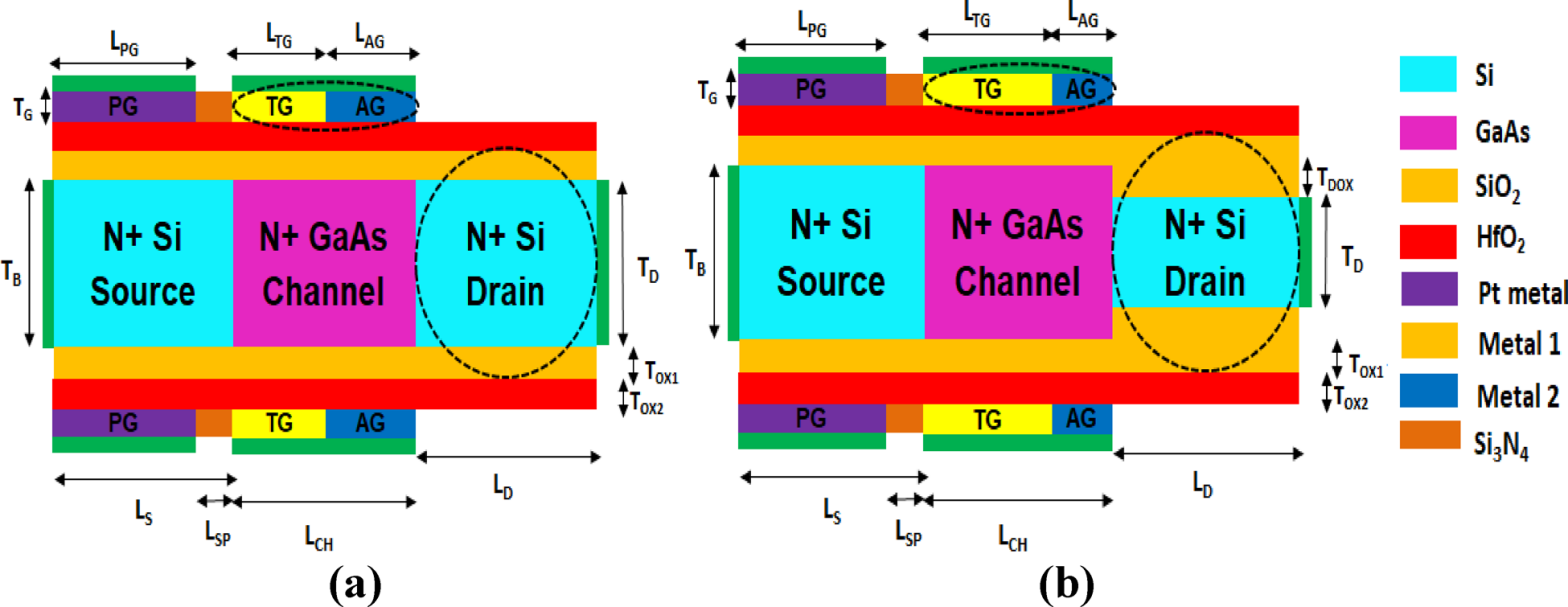
(**a**) Cross sectional view of HJ-DD-UTFET (symmetric design). (**b**) Cross sectional view of A-SD-HJ-DD-UTFET (Asymmetric design).

The HJ-DD-UTFET features a symmetrical and uniform structure where the source, channel, and drain regions have the uniform thickness and both the Tunneling Gate (TG) and Auxiliary Gate (AG) are of equal length as depicted in Fig. 1a. While this design simplifies fabrication and ensures structural uniformity, it introduces several performance limitations compared to the proposed A-SD-HJ-DD-UTFET. The electrical characteristics of the proposed device has been reported in our previous study^63^ along with its biosensing performance for dengue NS1 protein detection. Unlike the symmetric design, the A-SD-HJ-DD-UTFET features a reduced drain region to reduce the OFF-state leakage current and an asymmetrical gate configuration featuring a 15 nm TG and a 5 nm AG given in the Fig. 1b which optimizes tunneling efficiency through better electrostatics and aids in suppressing leakage current respectively. A SiO_2_ interfacial layer present in-between the GaAs and HfO_2_ layer has proven to improve the surface stability, while HfO_2_ passivates the interface defects and reduces trap density^68–70^ and also the Interface Trap Charge (ITC) effects of the proposed device were analysed in^63^ proving that the proposed device is highly immune to ITCs. GaAs in the channel helps to boost RF performance by improving carrier mobility, reducing tunneling barriers and optimizing conduction band alignment for efficient charge transport than the conventional JLTFETs. Even though both the designs achieve almost the same ON-state current in the orders of 10^–4^ A/µm, the HJ-DD-UTFET suffers from significantly higher OFF-state leakage current, with an OFF current—four orders of magnitude greater than the asymmetric configuration (ie., 4.44 × 10^–13^ A/µm and 8.124 × 10^–17^ A/µm respectively) due to the equal source-drain thickness and lack of asymmetric gate scaling as it weakens electrostatic control, deteriorating the device linearity performance. This increased leakage degrades subthreshold performance, reduces transconductance efficiency and Cut-Off Frequency (f_T_), which negatively impacts RF performance. Furthermore, in dengue NS1 protein biosensing, the symmetric design’s weaker gate-channel modulation reduces sensitivity to biomolecular interactions due to a lower ON OFF current ratio, resulting in lower signal resolution and detection accuracy. This capability is crucial for applications in medical diagnostics, offering improved sensitivity and accuracy for detecting disease markers at early stages. The parameters, variables and values used in the proposed design are tabulated in the Table 1 below.

**Table 1:** Parameters, variables and values used in the proposed HJ-DD-UTFET and A-SD-HJ-DD-UTFET devices.

Parameter	Variables	Values (HJ-DD-UTFET) symmetric	Values (A-SD-HJ-DD-UTFET) asymmetric
Length of source, channel and drain	L_S_, L_CH_, L_D_	20 nm each	20 nm each
Thickness of body, drain and drain oxide	T_B_, T_D_, T_DOX_	10 nm, 10 nm, 0 nm (T_B_ = T_D_)	10 nm, 6 nm, 2 nm
Thickness of gate, SiO_2_, HfO_2_	T_G_, T_OX1_, T_OX2_	1 nm each	1 nm each
Length of PG, TG, AG, spacer	L_PG_, L_TG_, L_AG_, L_SP_	17 nm, 10 nm, 10 nm, 3 nm	17 nm, 15 nm, 5 nm, 3 nm
Uniform doping concentration	N_D_	1 × 10 ^19^ cm ^−3^	1 × 10 ^19^ cm ^−3^
Work function of PG, TG, AG	Φ_PG_, Φ_M1_, Φ_M2_	5.93 eV, 5.1 eV, 4.9 eV	5.93 eV, 5.1 eV, 4.9 eV

## Models used and simulation setup

**Fig. 2:**
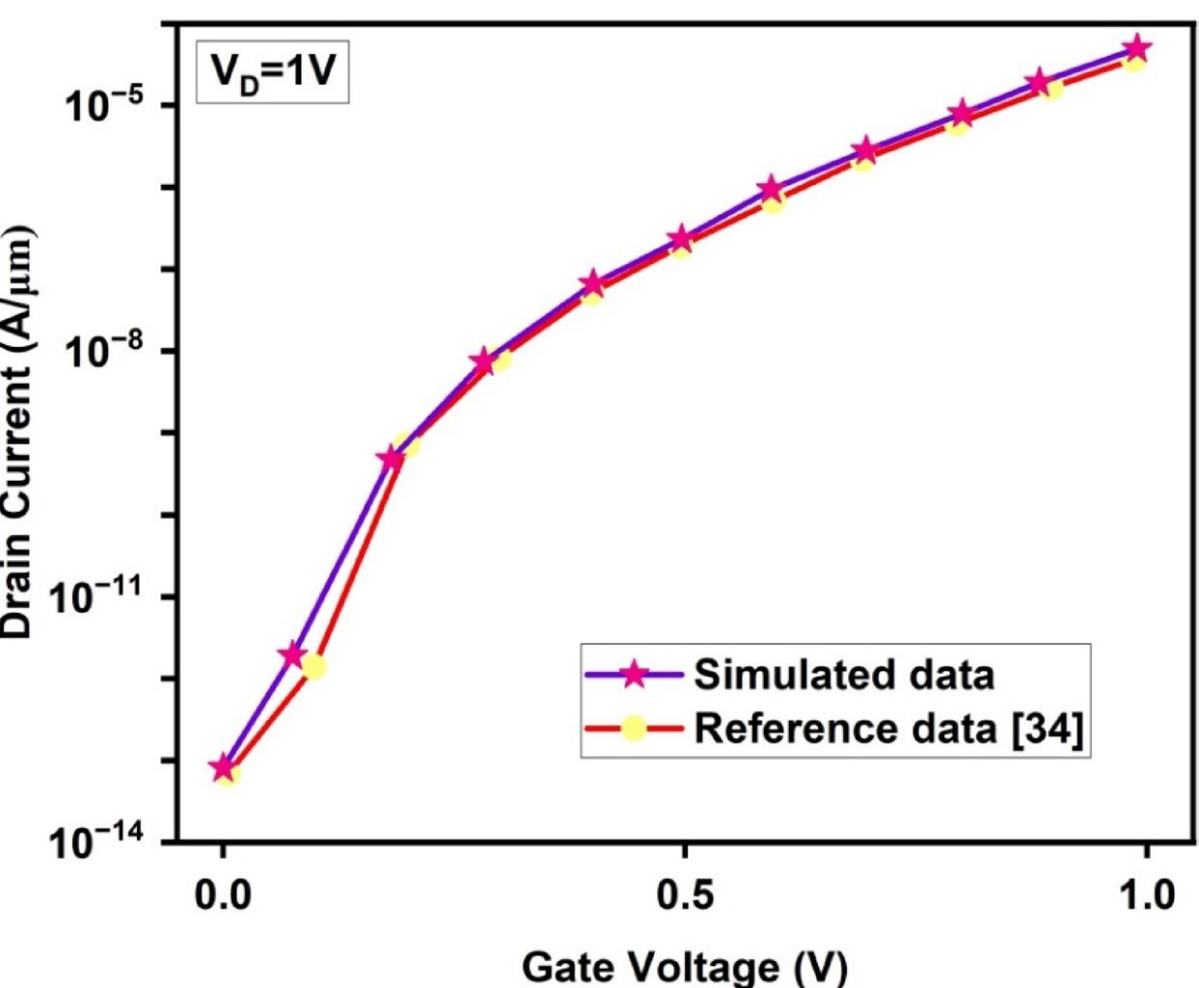
Calibration of TCAD simulation with a reference work having kept all the parameters same^34^.

**Fig. 3:**
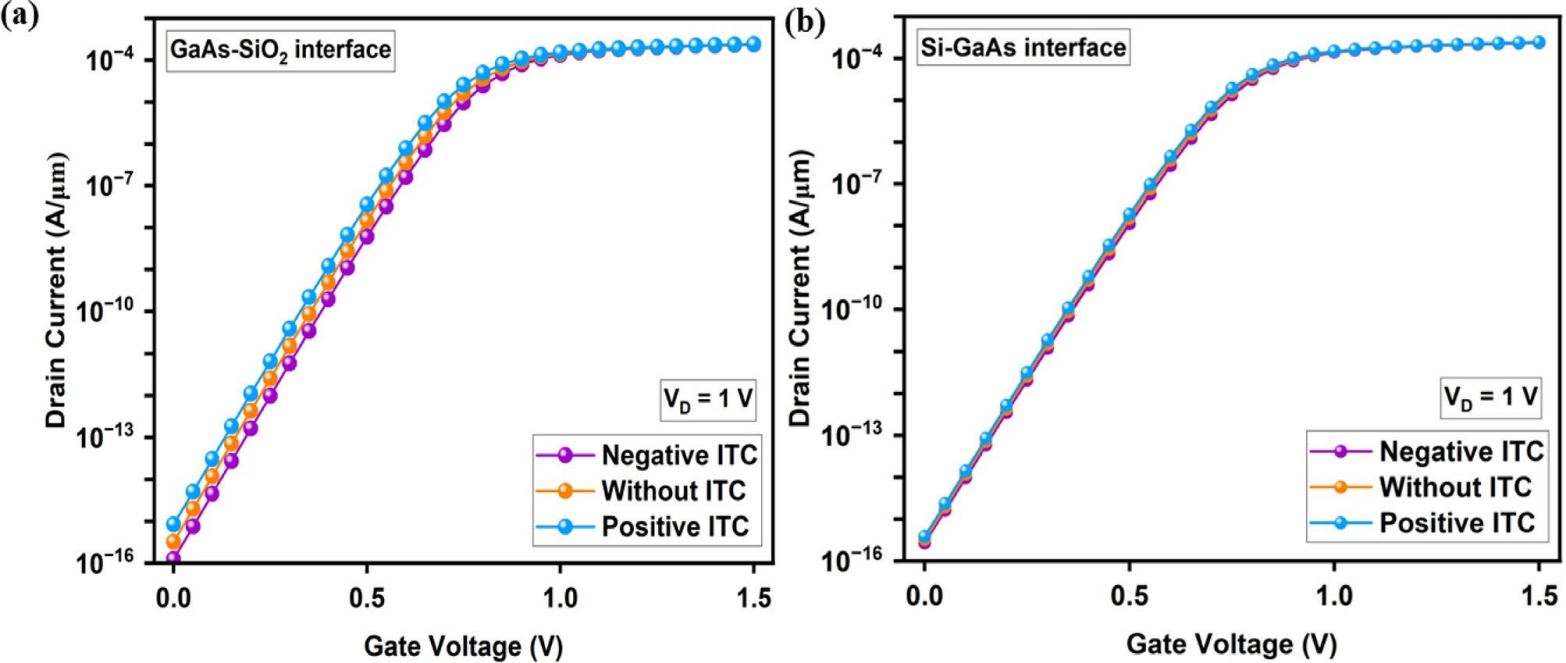
Effect of Interface Trap Charges on (**a**) GaAs-SiO_2_ interface. (**b**) Si-GaAs interface.

The experimental realization of the proposed asymmetric JLTFET structure has not yet been reported in the literature. Therefore, Sentaurus TCAD simulations were calibrated using an initially reported symmetric JLTFET data^34^ as shown in Fig. 2. The close agreement between the transfer characteristics validates the simulation framework, where structural and material parameters are kept constant. Additionally, the simulation incorporates modeling parameters and physical effects based on our prior UTFET work^63^, ensuring continuity in methodology and device representation. The simulation uses a comprehensive set of physical models adopted from^34^ and^63^ to accurately capture key transport mechanisms in nanoscale TFETs. These include the nonlocal Band-to-Band Tunneling (BTBT) model for lateral tunnelling which accurately captures the spatial separation of the tunneling path, especially critical for heterojunctions, Band Gap Narrowing (BGN) for high doping effects, Shockley–Read–Hall (SRH) and Auger Recombination models for impurity and trap-induced carrier recombination. Quantum Confinement (QC) effects for ultra-thin 10 nm channel are included, while Schenk’s Trap Assisted Tunneling (TAT) model accounts for defect-mediated tunneling. Interface trap charges at GaAs/SiO₂ and GaAs/Si interfaces were modelled using uniform fixed charges (± 1 × 10^12^ cm^-2^) as given in Fig. 3a and b. The device showed negligible OFF state performance degradation, indicating strong immunity to ITCs^58,71,72^.

To model carrier mobility degradation in high-field regions, the concentration and field-calibration dependent (Lombardi CVT) mobility model is employed to describe the device behaviour at a constant Voltage and Temperature. Gate leakage current is neglected based on the assumption of a high-κ metal gate stack. Interface Trap Charges (ITCs) are incorporated to realistically reflect device behavior under biosensing conditions and analog performance degradation. Recent studies^73–75^ have demonstrated that ITCs significantly affect subthreshold slope, transconductance (g_m_), and linearity in TFET-based analog and RF applications. Material parameters for the GaAs/Si heterojunction including bandgap, dielectric constant, electron affinity, and effective masses are taken from experimentally validated datasets and literature. Specifically, the bandgap energy is modelled using the Eq. 1 below with parameters: for silicon, E_G_(0) = 1.12416 eV, α = 4.73 × 10^–4^ eV/K, and β = 636 K; and for GaAs, E_G_(0) = 1.519 eV, α = 5.405 × 10^–4^ eV/K, and β = 204 K^76^.

1 $$E_{G} \left( T \right) = E_{G} \left( 0 \right) - \frac{{\alpha T^{2} }}{T + \beta }$$

Fermi-level pinning (FLP) effects at the GaAs/Si interface are implicitly considered via work function adjustment and interface trap modeling, avoiding artificial barrier lowering^75,77^. Furthermore, the Debye length, critical in nanoscale electrostatics, is accounted for by choosing device body and gate oxide thicknesses to ensure strong gate control and minimal SCEs. This design approach aligns with accepted modeling practices for ultra-thin body TFETs^58,72,78^.

**Fig. 4:**
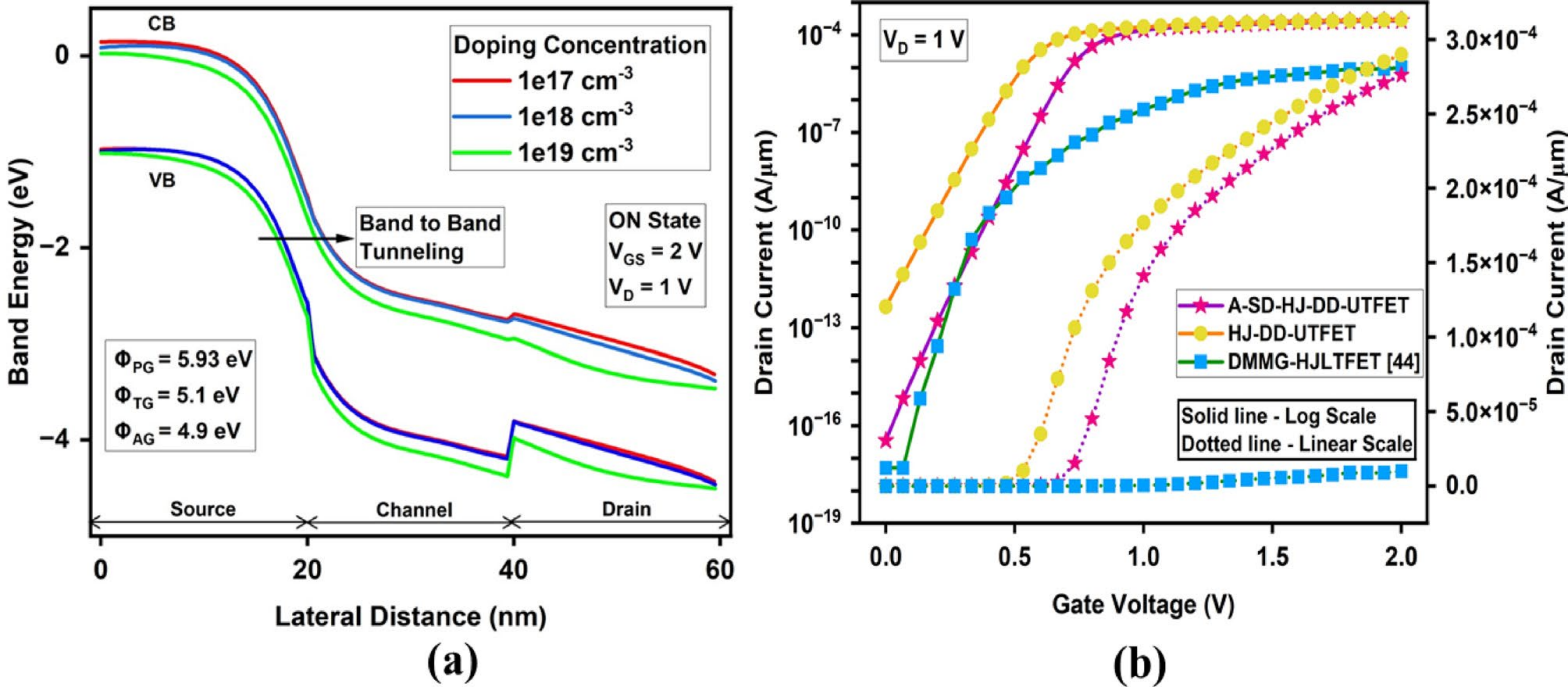
(**a**) Energy band diagram of the proposed device for various doping concentration (10^17^–10^19^ cm^-3^). (**b**) Transfer characteristics comparison (Linear & Log scale).

The electrical parameters and optimized work function choices of the proposed designs are given in detail in our previous biosensor work^63^, where the suitability of these parameters for biosensing applications were demonstrated. The energy band diagram is plotted in the Fig. 4a with variations in uniform doping concentrations (N_D_) such as 1 × 10^17^, 1 × 10^18^, 1 × 10^19^ cm^-3^, while 1 × 10^19^ cm^-3^ concentration showing the best performance, characterized by higher ON state current, lower OFF state leakage current, improved SS and enhanced band bending. The BTBT phenomenon is also depicted in the Fig. 4a. This is attributed to better tunneling efficiency and superior electrostatic control, which lead to more efficient charge carrier transport. These improvements were observed consistently in both symmetric and asymmetric designs. However, this paper primarily focuses on a comparative investigation of the RF and linearity performance of HJ-DD-UTFET and A-SD-HJ-DD-UTFET devices, as well as the RF performance of A-SD-HJ-DD-UTFET biosensor. For analyzing the RF performance of the proposed symmetric and asymmetric devices, in addition to the above models, the Velocity Saturation model is also employed to manage carrier velocity saturation in high electric fields for accurately simulating the device performance, particularly in short channel devices where these effects become more prevalent. Furthermore, the Lombardi mobility model (CVT) is employed to account for surface roughness, phonon scattering, and Coulomb interactions, ensuring accurate mobility estimation crucial for RF performance analysis.

In asymmetric A-SD-HJ-DD-UTFET, the very lower OFF state leakage current is a key feature that significantly enhances its performance. When no gate bias is applied (V_GS_ = V_D_ = 0 V), the tunneling barrier at Si/GaAs interface becomes sufficiently wide to restrict the flow of minority carriers, ensuring minimal ambipolar current. As a result, the OFF-state current (I_OFF_) remains extremely low. When applying the gate voltage (V_GS_ = 2 V, V_D_ = 1 V), the narrowing of the tunneling barrier at the Si/GaAs source-channel interface allows an increased number of carriers to tunnel through, leading to a sharp rise in ON state current. Importantly, the leakage current, which contributes to power dissipation and reduce device efficiency, remains very low due to the nature of the tunneling process and the selective control over carrier injection.

The comparative analysis of A-SD-HJ-DD-UTFET, HJ-DD-UTFET, and DMMG-HJLTFET (Dual Metallic Material Gate Heterostructure JLTFET)^44^ in the Fig. 4b highlights the superior performance of the asymmetric design due to its advanced structural optimizations. The A-SD-HJ-DD-UTFET achieves an ON-state current (I_ON_) of 2.305 × 10^−4^ A/μm, which is slightly higher than the HJ-DD-UTFET’s 2.01 × 10^−4^ A/μm and relatively higher than the DMMG-HJLTFET’s 9.04 × 10^−6^ A/μm. Also, the A-SD-HJ-DD-UTFET excels in the I_ON_/I_OFF_ratio, achieving an impressive 2.83 × 10^12^, far surpassing the HJ-DD-UTFET’s 4.52 × 10^8^. This is primarily due to the A-HJ-DD-UTFET’s ultra-low OFF state current (I_OFF_) of approximately 8.124 × 10^−17^ A/μm, achieved through asymmetric design and heterojunction bandgap engineering. The threshold voltage of the DMMG-HJLTFET is also found to be more than 1 V which results in a degraded RF performance. Comparison of electrical characteristics of various JLTFET devices are tabulated in below Table 2.

**Table 2:** Comparison of electrical characteristics of various JLTFET devices.

Device name	Gate/Channel length (nm)	Control gate work function (eV)	V_D_ (V)	V_G_ (V)	ON current (A/µm)	OFF current (A/µm)	SS (mV/dec)	Refs.
DMCG-JLTFET	20	4.1, 4.8, 4.1	1	1	~ 10^–7^	~ 10^–17^	-	^40^
DDG-GOHJLTFET	20	4.5	1.5	1.5	1.36 × 10^–4^	1.81 × 10^–16^	30.8	^79^
HMG-HJLTFET	20	4.1, 4.5	1.5	1.5	1.06 × 10^–4^	~ 10^–16^	26.2	^46^
DO-DMG-JL-TFET	50	4.7, 4.6	1	1.2	7.6 × 10^–6^	2.8 × 10^–17^	36.12	^47^
DO-ED-JL-TFET	50	4.72	0.5	1	~ 10^–5^	~ 10^–16^	42.7	^55^
P + pocket HJLTFET	30	4.7	1	1	1.80 × 10^–4^	~ 10^–11^	42.3	^43^
VDL-DG-JLFET	20	4.9	0.1	1	~ 10^–3^	~ 10^–13^	68	^50^
DMS-CP-JL-TFET	50	4.7	0.5	1	7.63 × 10^–7^	2.03 × 10^–17^	12.74	^45^
SON-ED-JLTFET	20	4.17	1	1.2	6.11 × 10^–7^	3.54 × 10^−18^	15.133	^56^
DMG-HJLTFET	20	4.5	1.5	1.5	8.85 × 10^–5^	2.90 × 10^–16^	13.8	^80^
DMG-H-JLTFET	30	4.7, 4	1	1.2	~ 10^–3^	7.80 × 10^–14^	29	^39^
HJ-DD-UTFET	20	5.1, 4.9	1	2	2.01 × 10^–4^	4.44 × 10^–13^	15.57	This work
A-SD-HJ-DD-UTFET (Reported work)	20	5.1, 4.9	1	2	2.3 × 10^–4^	8.12 × 10^–17^	14.56	

DMCG-JLTFET - Dual Material Control Gate JLTFET, DDG-GOHJLTFET - Dual Dielectric Gate-Gate Overlap Heterostructure JLTFET, HMG-HJLTFET - Hetero Metal Gate-Heterostructure JLTFET, DO-DMG-JL-TFET - Dual Oxide Dual Material Gate JLTFET, DO-ED-JL-TFET - Dual Oxide Electrically Doped JLTFET, VDL-DG-JLFET - Vertical Dopingless Double Gate FET), DMS-CP-JL-TFET - Dual Meta Strip Charge Plasma based JLTFET, SON-ED-JLTFET - Silicon On Nothing Electrostatically Doped JLTFET, DMG-HJLTFET - Dual Metal Gate Hetero-material JLTFET.

## Analog/RF figure of merits

Transconductance (g_m_) is a key parameter in TFETs that describes how effectively a device can control its output current (I_D_) in response to changes in the gate to source voltage (V_GS_)^40^. It is mathematically defined as the derivative of the I_D_ with respect to the V_GS_ at a constant drain voltage (V_D_) and is given in Eq. 2 as,

2 $$g_{m} = \frac{{\partial I_{d} }}{{\partial V_{GS} }}$$

This parameter is crucial for the performance of transistors, particularly in RF and analog applications, because it dictates the ability of the device to amplify signals and drive current. A higher transconductance translates to better current driving capability and higher gain, which are essential for efficient amplification and high frequency performance.

**Fig. 5:**
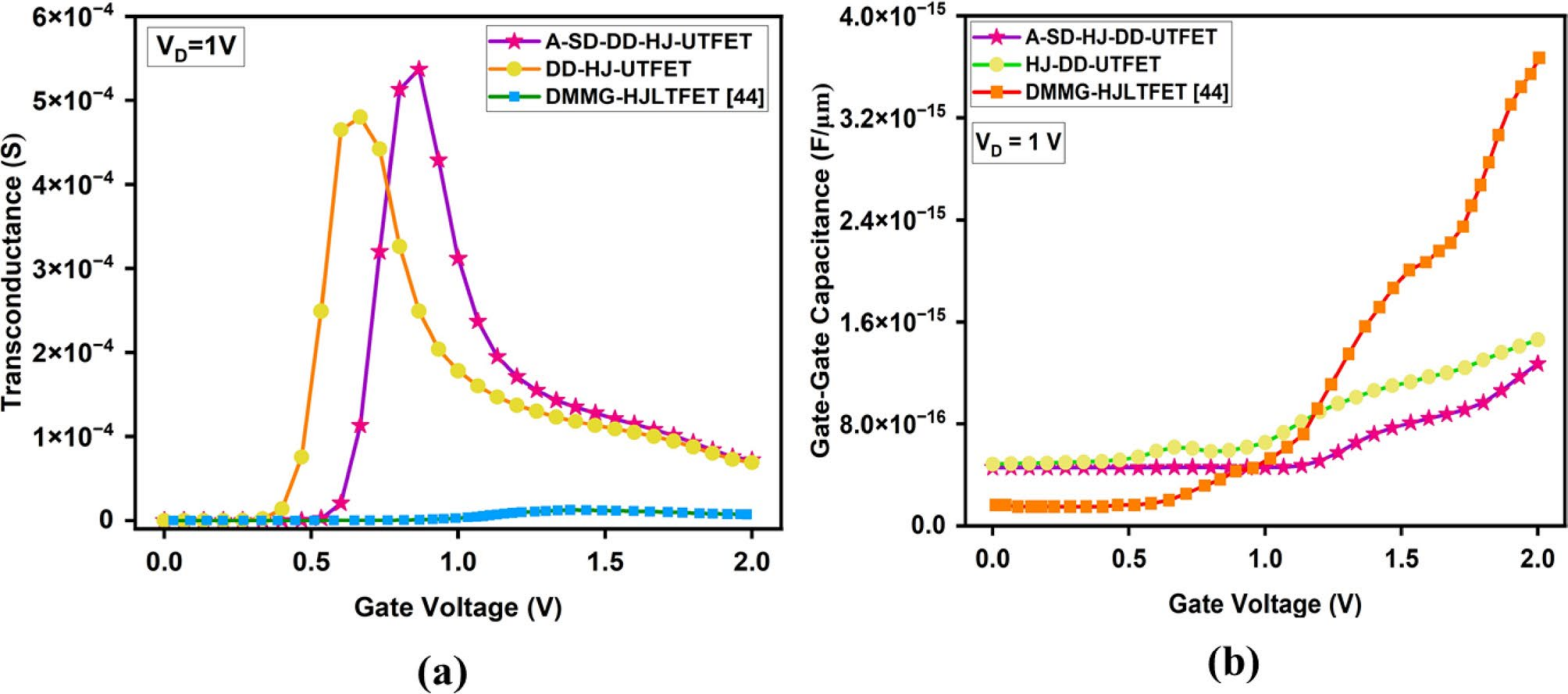
Comparative plot of (**a**) Transconductance (g_m_) (**b**) Gate to Gate Capacitance/ Total Capacitance (C_gg_).

For RF applications, the transconductance is directly linked to the efficiency and linearity of the amplification process. Here, from the Fig. 5a, it is evident that the proposed A-SD-HJ-DD-UTFET demonstrates maximum transconductance (g_m_) of 5.365 × 10^−4^ S, surpassing the HJ-DD-UTFET (4.8 × 10^−4^ S) and the DMMG-HJLTFET (1.26 × 10^−5^ S), as a result of asymmetric and optimized gate lengths with proper WFs to ensure superior channel control which is crucial for high gain and efficient signal amplification in RF applications.

The gate-gate capacitance/Total capacitance (C_gg_ = C_gs_ + C_gd_) plays a critical role in analyzing the RF performance. Lower C_gg_ enhances both GFP and GTFP by minimizing the capacitive delay, thereby increasing the speed of signal amplification. However, C_gg_ must be optimized, as excessive reduction could compromise the electrostatic control and transconductance (g_m_). Parasitic capacitances, particularly C_gd_, significantly affect RF performance by introducing feedback and reducing isolation between the input and output, which limits the frequency response. Specifically, the asymmetry introduces a reduced effective drain overlap and channel thickness on the drain side, decreasing the overlap area contributing to C_gd_. Reducing C_gd_ directly reduces the effective capacitance (C_eff_) under the Miller effect, which in turn enhances switching speed and frequency characteristics of the device. Due to the Miller effect, C_gd_ is effectively amplified by the voltage gain, further degrading the bandwidth and stability of the device. The advanced A-SD-HJ-DD-UTFET design reduces the effective gate-to-drain overlap and drain thickness, thereby suppressing C_gd_ and mitigating the Miller effect through dual-dielectric and heterostructure engineering, ensuring superior RF metrics. From Fig. 5b, it is evident that the proposed A-SD-HJ-DD-UTFET exhibits the lowest gate-to-gate capacitance (C_gg_) in the order of 10^–16^ F/µm compared to the other devices. This reduction is primarily due to the asymmetric gate design and thinner drain, which minimize gate-to-channel and gate-to-drain overlap. The optimized electrostatics lead to lower parasitic capacitance, enhancing its suitability for high-frequency, low-power applications.

**Fig. 6:**
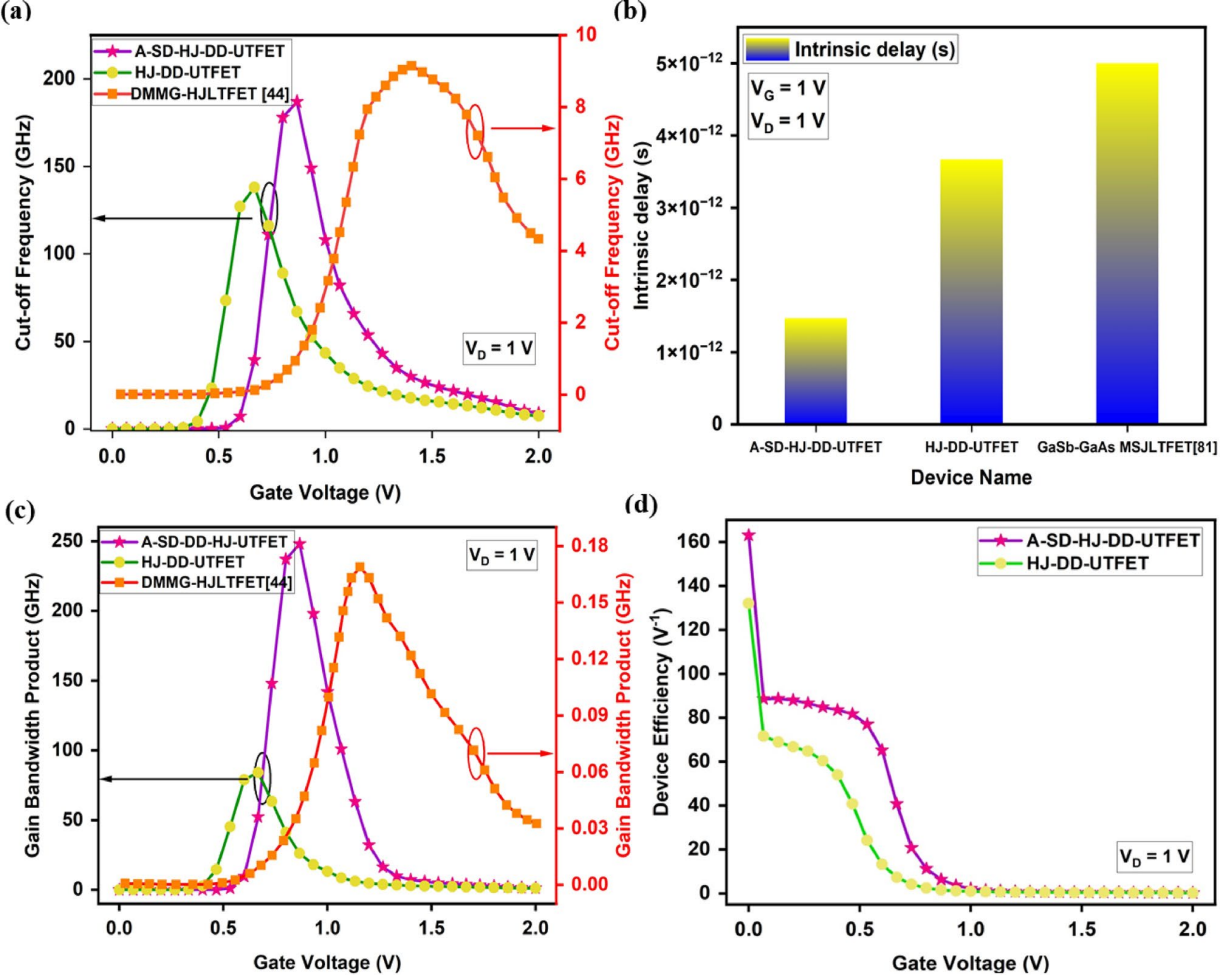
Comparison of (**a**) Cut-off frequency (f_T_) (**b**) Transit time (τ) (**c**) Gain bandwidth product (GBP) (**d**) Device efficiency (TGF) of the device.

Cut-off frequency (f_T_) is another essential parameter in RF and analog applications. It refers to the frequency at which the current gain of the device drops to 1 (or -3 dB in the logarithmic scale). It provides a measure of how fast the device can operate and is linked to the transition from the active region to the cut-off region in a transistor. From the Fig. 6a, the Cut Off frequency (f_T_) further underscores the dominance of A-SD-HJ-DD-UTFET, reaching a maximum value of 187 GHz, a 35.5% improvement over the HJ-DD-UTFET (138 GHz) and significantly very much higher than the DMMG-HJLTFET (9.17 GHz). Since f_T_ is directly proportional to g_m_, a higher g_m_ in the device naturally leads to a higher f_T_. This means that the device can operate effectively at higher frequencies, and it ensures faster signal processing in RF applications. Overall, the asymmetric A-SD-HJ-DD-UTFET’s structural innovations, including asymmetric gate control, dual dielectrics for lower gate capacitance, and a bandgap engineered Si/GaAs interface, result in lower I_OFF_, a higher I_ON_/I_OFF_ ratio, improved g_m_ and enhanced f_T_, establishing it as the superior choice for low power, analog, and RF applications. f_T_ is calculated using the formula given in^55^ and represented in Eq. 3 as

3 $$f_{T} = \frac{{g_{m} }}{{2\pi C_{GS} \left( {\sqrt {1 + 2\frac{{C_{GD} }}{{C_{GS} }}} } \right)}} = \frac{{g_{m} }}{{2\pi \left( {C_{GS} + C_{GD} } \right)}}$$

Also, the transit time (τ) that determines the carrier transport delay from source to drain is crucial in RF applications. A shorter transit time ensures faster signal transmission, higher switching speeds, and improved cut-off frequency (f_T_). This directly enhances TFET performance in high-frequency circuits, making it ideal for RF and biosensing applications. From the Fig. 6 (b), it is clear that the proposed A-SD-HJ-DD-UTFET achieves the lowest transit time of 1.47 × 10^–12^ s, which is significantly lower than the HJ-DD-UTFET (3.67 × 10^–12^ s) and the GaSb GaAs Metal Strip (MS)JLTFET (5 × 10^–12^ s)^81^. This reduction in transit time is attributed to the stronger electric field at the tunneling junction, which facilitates rapid charge carrier transport, ensuring superior high-frequency operation. Transit time is represented in Eq. 4 as,

4 $$\tau = \frac{1}{{2\pi f_{T} }}$$

Next, the Gain Bandwidth Product (GBP) is a crucial analog and RF figure of merit that reflects a device’s ability to maintain voltage gain across a defined frequency range, typically up to the 3 dB bandwidth. Unlike GFP, which involves cut-off frequency, GBP emphasizes the usable bandwidth for effective amplification. A higher transconductance enables stronger current drive, which directly improves GBP and extends the biosensor’s operational frequency range. A high GBP ensures efficient operation in wireless communication, high-speed signal processing, and biosensing**.** The A-SD-HJ-DD-UTFET demonstrates superior gain-bandwidth product due to its optimized g_m_ and C_gd_, making it highly suitable for high-frequency RF applications. The incorporation of Si-GaAs heterojunction significantly enhances tunneling efficiency**,** leading to a higher g_m_, which directly improves GBP. From the Fig. 6c demonstrates that the proposed A-SD-HJ-DD-UTFET attains an exceptionally higher GBP of 248 GHz at 0.86 V, significantly outperforming the HJ-DD-UTFET (84.2 GHz at 0.66 V) and the DMMG-HJLTFET (0.168 GHz at 1.15 V). These advantages collectively position the A-SD-HJ-DD-UTFET to be the superior device candidate for RF and analog applications**.**

The gain-bandwidth product (GBP) for a gain of 10 is defined by the equation as

5 $$GBP = \frac{{g_{m} }}{{2\pi \left( {10C_{gd} } \right)}}$$

The transconductance generation factor (TGF), given by

6 $$TGF = \frac{{g_{m} }}{{I_{D} }}$$

TGF measures the device’s efficiency in converting input voltage variations into output current, with a higher value indicating better power efficiency and signal amplification. From the Fig. 6d it is evident that the A-SD-HJ-DD-UTFET achieves a superior device efficiency, outperforming its counterparts due to optimized tunneling and enhanced carrier transport. The Transconductance Frequency Product (TFP), defined as

7 $$TFP = TGF \times f_{T} = { }\frac{{g_{m} }}{{I_{D} }} \times f_{T}$$

**Fig. 7:**
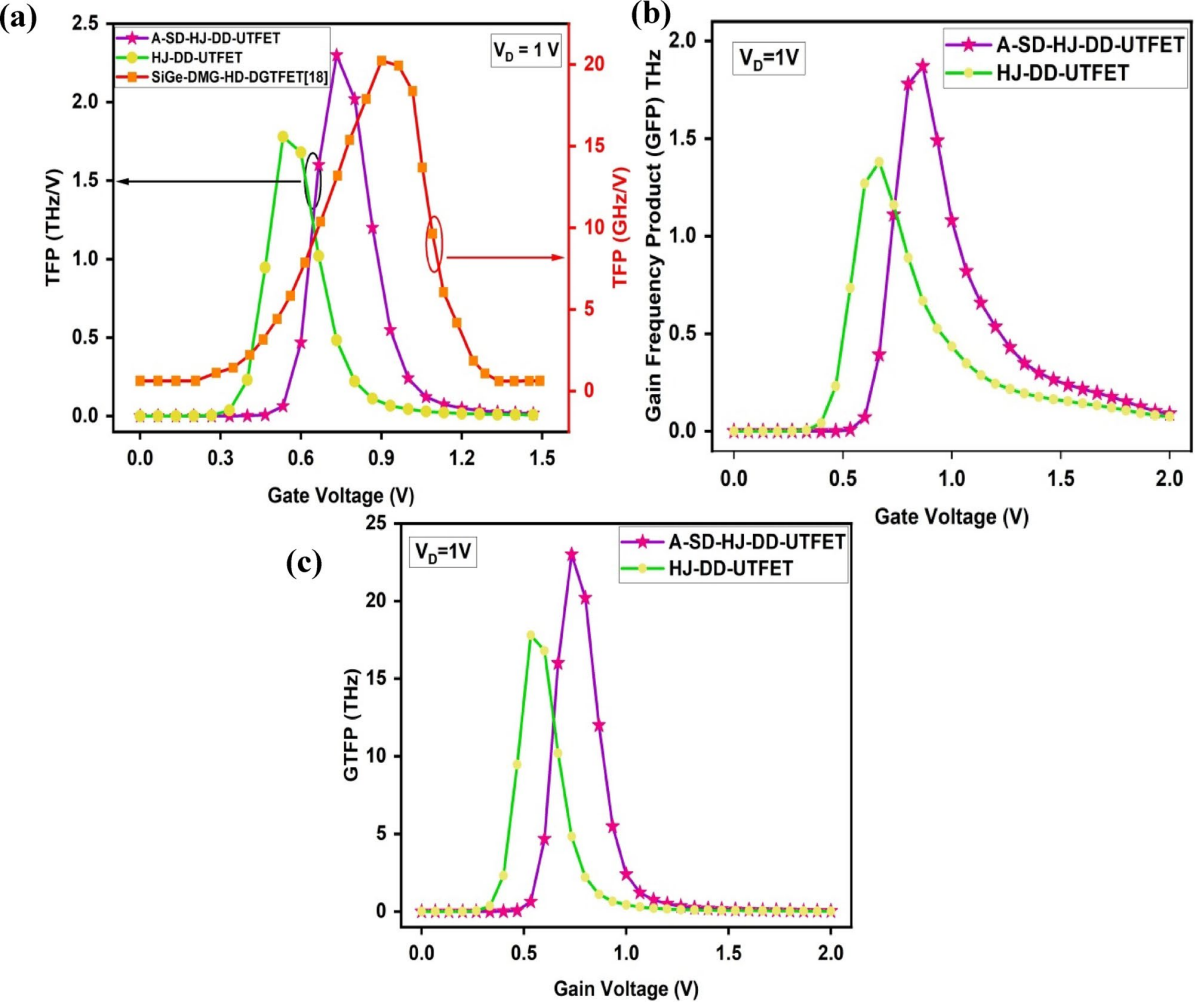
Comparison of (**a**) Transconductance frequency product (TFP) (**b**) Gain frequency product (**c**) Gain transconductance frequency product (GTFP) of the device.

TFP represents the high-frequency amplification efficiency of the device. It can be observed from the Fig. 7a that the A-SD-HJ-DD-UTFET exhibits a superior TFP of 2.3 THz at 0.733 V, outperforming the HJ-DD-UTFET (1.78 THz at 0.533 V) and the SiGe-DMG-HD-DGTFET (20.24 GHz at 0.9 V)^18^. This enhancement is driven by the higher transconductance (g_m_) and Cut-Off frequency (f_T_) in the A-SD-HJ-DD-UTFET, which result from its heterojunction architecture, increased tunneling efficiency, and suppressed parasitic effects.

The Gain Frequency Product (GFP) represents the product of a device’s voltage gain (A_V_) and its cut-off frequency (f_T_**)**. It reflects the combined impact of amplification capability and frequency response, serving as a key figure of merit for analog and RF performance. GFP is given by

8 $$GFP = A_{V } \times f_{T} = 10 \times \frac{{g_{m} }}{{2\pi \left( {C_{gg} } \right)}}$$

where g_m_ is the transconductance and C_gg_ is the total gate capacitance. As seen in the Fig. 7b the proposed A-SD-HJ-DD-UTFET achieves a peak GFP of 1.87 THz at 0.866 V, significantly higher than the HJ-DD-UTFET’s 1.38 THz at 0.66 V. This enhancement is due to improved tunneling efficiency and a steeper subthreshold slope, resulting in higher g_m_ while maintaining a moderate increase in C_gg_.

Similarly, The Gain Transconductance Frequency Product (GTFP) is defined as the product of transconductance (g_m_) and the Gain Frequency Product (GFP) and it represents the combined effect of transconductance and frequency response on device performance. GTFP is defined as:

9 $$GTFP = TGF \times GFP = \frac{{g_{m} }}{{I_{D} }} \times 10 \times \frac{{g_{m} }}{{2\pi \left( {C_{gg} } \right)}}$$

From the GTFP graph given in Fig. 7c it is evident that the proposed A-SD-HJ-DD-UTFET attains a peak GTFP of 23 THz at 0.733 V, outperforming the HJ-DD-UTFET’s 17.8 THz at 0.533 V. This improvement is attributed to the optimized asymmetric source/drain design and advanced Si/GaAs heterostructure, which reduces the parasitic capacitances resulting in a higher g_m_. The comparison of various analog/RF Figure of Merits (FOMs) of the proposed device with other JLTFETs are reported in the below Table 3 and it can be found that the proposed A-SD-HJ-DD-UTFET device offers superior RF FOMs than the other JLTFETs.

**Table 3:** Comparison of analog/RF Figure of Merits (FOMs) of various JLTFET devices.

Device name	Materials used	I_ON_/I_OFF_ ratio	g_m_ (µS)	f_T_ (GHz)	GBP (GHz)	Device efficiency (TGF) V^-1^	TFP (THz/V)	Refs.
DMCG-JLTFET	Si	~ 10^10^	~ 1.05	~ 0.79	0.085	~ 75	~ 0.036	^40^
DO-DMG-JL-TFET	Si	2.7 × 10^11^	40.5	2	0.2	-	-	^47^
DO-ED-JL-TFET	Si	3.32 × 10^11^	~ 44	2.5	0.25	-	-	^55^
P + pocket HJLTFET	Ge/Si	1.80 × 10^7^	170	100	118	-	-	^43^
SON-ED-JLTFET	Si	1.72 × 10^11^	3.227	-	1	~ 90	0.055	^56^
DMG-HJLTFET	InAs/GaAs	3.1 × 10^11^	370	-	-	-	-	^80^
DMMG-HJLTFET	Ge/Si/Si + GaAs	1.56 × 10^13^	11.1	9.17	0.17	-	-	^44^
DMG-H-JLTFET	Ge/Si	1.2 × 10^10^	280	-	-	-	-	^39^
HJ-DD-UTFET	Si/GaAs	4.52 × 10^8^	480	138	84.2	132	1.78	This work
A-SD-HJ-DD-UTFET	Si/GaAs	2.83 × 10^12^	536	187	248	163	2.30	

DMCG-JLTFET - Dual Material Control Gate JLTFET, DO-DMG-JL-TFET - Dual Oxide Dual Material Gate JLTFET, DO-ED-JL-TFET - Dual Oxide Electrically Doped JLTFET, SON-ED-JLTFET - Silicon on Nothing Electrostatically Doped JLTFET, DMMG-HJLTFET - Dual Metallic Material Gate Heterostructure JLTFET, DMG-HJLTFET - Dual Metal Gate Hetero-material JLTFET.

## Linearity analysis

Linearity in a TFET device is essential in analog and RF applications to minimize distortion and ensure accurate signal amplification. These parameters are evaluated through g_mn_, VIP2, VIP3, IIP3, IIP3 (dBm), IMD3, IMD3 (dBm), and the 1 dB compression point.

**Fig. 8:**
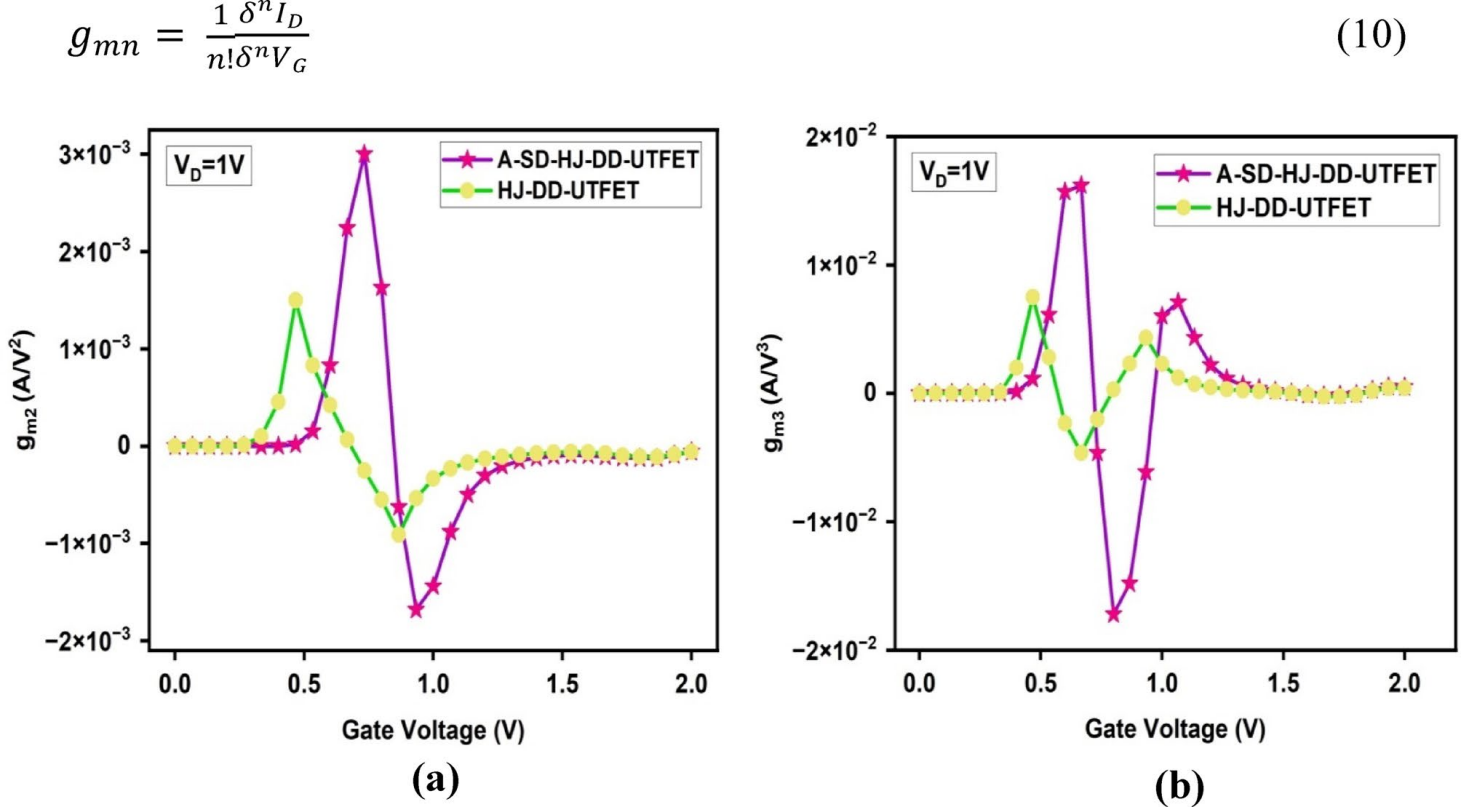
Comparison of (**a**) Second order transconductance (g_m2_) (**b**) Second order transconductance (g_m3_) of the device.

The second and third order transconductance is essential in a device to evaluate its higher-order nonlinearity and to assess its performance in analog and RF applications, especially in terms of gain, linearity, and frequency response. They are evaluated through the below Eq. 10 and represented in the Fig. 8a and b respectively. These graphs show lower g_m2_ and g_m3_ values for the A-SD-HJ-DD-UTFET in comparison with HJ-DD-UTFET, highlighting the reduced higher-order nonlinearity and improved linearity, which are crucial for enhanced gain, linearity, and frequency response in analog and RF applications.

10 $$g_{mn} = \frac{1}{n!}\frac{{\delta^{n} I_{D} }}{{\delta^{n} V_{G} }}$$

The Voltage Intercept Point 2 (VIP2) is a crucial parameter that quantifies the second-order nonlinearity of a TFET device, which directly affects even-order distortion products in RF circuits. It is defined as the extrapolated point where the fundamental signal and second-order intermodulation distortion components intersect. Mathematically, VIP2 is represented in Eq. 11 as

11 $$VIP2 = 4 \times \frac{{g_{m1} }}{{g_{m2} }}$$

where g_m1_ represents the first-order transconductance and g_m2_ is the second-order transconductance derivative^41,80^.

**Fig. 9:**
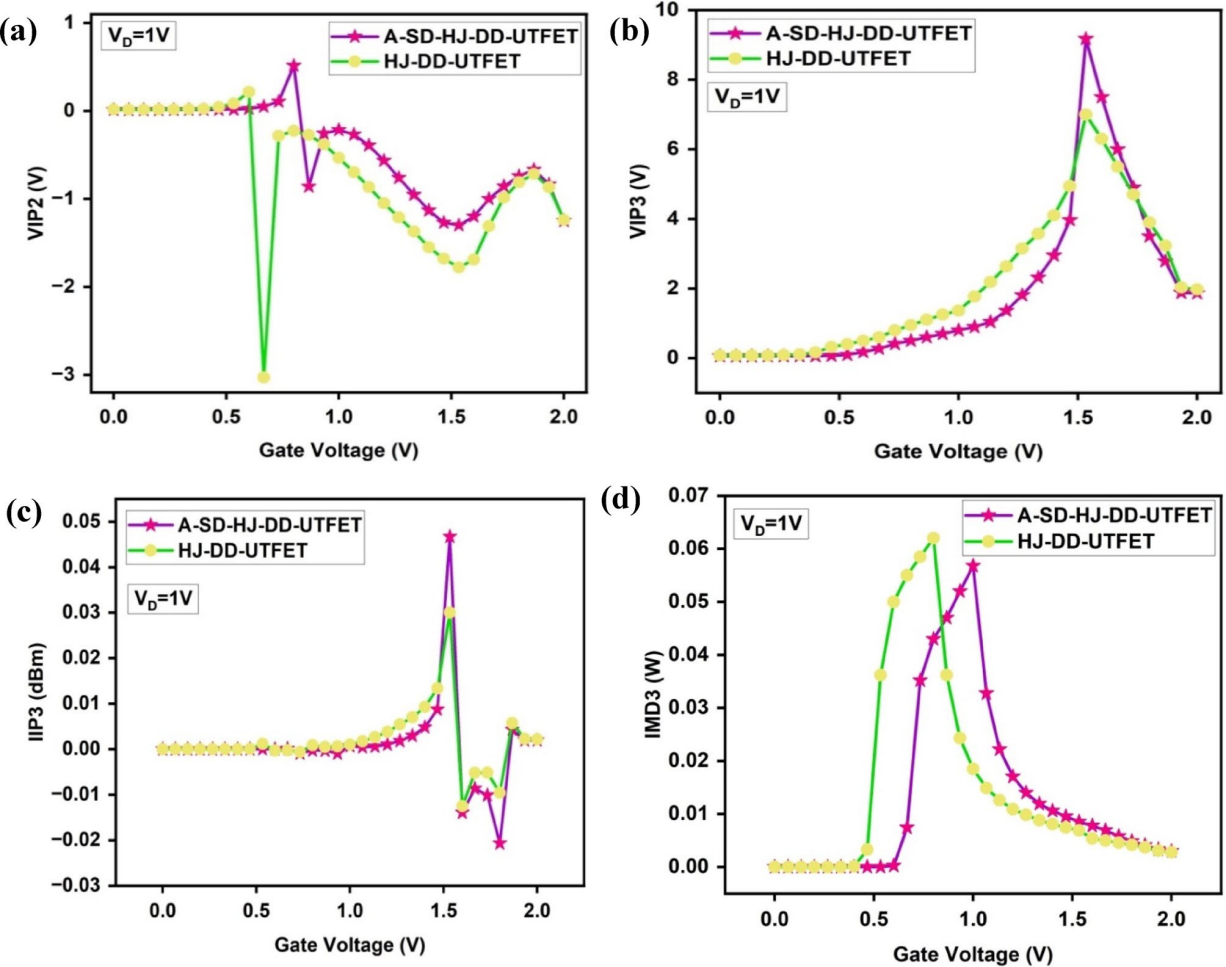
Comparison of (**a**) Voltage Intercept Point 2 (VIP2) (**b**) Voltage Intercept Point 3 (VIP3) (**c**) Input Intercept Point 3 (IIP3) (**d**) Third order Intermodulation Distortion (IMD3).

As shown in Fig. 9a, both devices exhibit stable VIP2 values at lower gate voltages, indicating minimal second-order nonlinearity. A sharp peak emerges near V_G_ ≈ 0.9 V, more pronounced for the A-SD-HJ-DD-UTFET, due to enhanced electrostatic gate control and abrupt increase in carrier tunneling at the onset of strong inversion. This leads to **a** higher first-order transconductance (g_m1_) and lower second-order transconductance (g_m2_), resulting in improved VIP2. A higher VIP2 indicates stronger immunity to second-order intermodulation distortion, which is vital for maintaining linearity in RF systems. The post-peak oscillations are attributed to nonlinear charge redistribution and variation in electric field across the channel with increasing V_G_^82,83^.

The Voltage Intercept Point 3 (VIP3) is an essential metric that determines the third-order linearity of the transistor, influencing intermodulation distortion in RF circuits. It is defined as the extrapolated input power level at which the fundamental signal and third-order intermodulation products intersect. The mathematical expression for VIP3 is:

12 $$VIP3 = \sqrt {24 \times \frac{{g_{m1} }}{{g_{m3} }}}$$

where g_m3_ is the third-order transconductance derivative. A higher VIP3 value indicates reduced third-order distortion. From the Fig. 9b, a steady increase in VIP3 with increasing gate voltage is observed, peaking before declining. The asymmetric A-SD-HJ-DD-UTFET exhibits a higher VIP3 than the symmetric counterpart, suggesting superior suppression of third-order distortion due to optimized gate control over the channel and reduced higher-order nonlinearity. The peak occurs because g_m1_ initially dominates over g_m3_, but as the gate voltage continues to rise, g_m3_ increases due to higher order charge interactions, leading to a decline in VIP3. A higher VIP3 is advantageous in RF applications as it enhances system linearity, reducing unwanted intermodulation products that degrade signal quality.

The Input Intercept Point 3 (IIP3) is a key parameter used to evaluate the third-order input-referred distortion in TFETs. It is defined as:

13 $$IIP3 = \frac{2}{3} \times \frac{{g_{m1} }}{{g_{m3} \times R_{S} }}$$

where R_S_ is the source resistance. A higher IIP3 suggests improved linearity, making the device more suitable for high frequency applications.

In Fig. 9c, the IIP3 parameter remains nearly flat for lower gate voltages and rises steeply near V_G_ ≈ 1.5 V, followed by fluctuations. The A-SD-HJ-DD-UTFET exhibits a higher peak IIP3 than the symmetric counterpart, indicating superior third-order linearity. This improvement results from effective electric field shaping in the drain region, which lowers g_m3_ while maintaining a strong g_m1_, thus reducing third-order intermodulation distortion (IMD3). The post-peak fluctuations indicate dynamic shifts in charge transport and field distribution under high gate bias, which slightly destabilize linearity. IIP3 in dBm is expressed as

14 $$IIP3\left( {{\text{dBm}}} \right) = 10 \log_{10} \left( {IIP3} \right) + 10$$

The Third order Intermodulation Distortion (IMD3) is a critical parameter in RF applications that measures the level of nonlinearity in a device. Lower IMD3 values indicate reduced signal distortion, which is crucial for high RF performance. The IMD3 can be represented in linear and logarithmic scales. The formula for calculating IMD3 in dBm is given as:

15 $$IMD3\left( {{\text{dBm}}} \right) = 10 \log_{10} \left( {IMD3} \right) + 10$$

where the linear IMD3 is calculated using the expression:

16 $$IMD3 = R_{S} \left[ {4.5 \times \left( {VIP3} \right)^{3} \times g_{m3} } \right]^{2}$$

The Fig. 9d shows the variation of linear IMD3 with gate voltage for the A-SD-HJ-DD-UTFET and HJ-DD-UTFET at V_D_ = 1 V. For ideal RF devices, the IMD3 should be as low as possible (both in linear and dBm scales) to minimize distortion.

From the Fig. 9d it can be observed that the A-SD-HJ-DD-UTFET demonstrates significantly lower IMD3 values compared to the HJ-DD-UTFET throughout the gate voltage range. Both devices exhibit a peak IMD3 near the transition to strong inversion, with the A-SD-HJ-DD-UTFET maintaining better suppression of nonlinearity beyond this region. The transition in this graph pinpoints that the A-SD-HJ-DD-UTFET structure minimizes distortion more effectively than its symmetric counterpart, particularly in the higher gate voltage region where RF devices often operate.

**Fig. 10:**
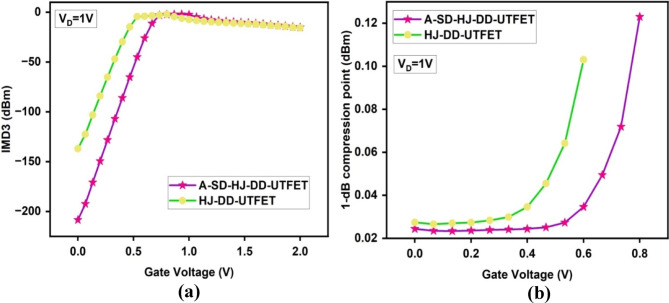
Comparison of (**a**) Third order Intermodulation Distortion (IMD3) (dBm) (**b**) 1-dB compression point of the device.

The Fig. 10a presents the IMD3 values on a logarithmic scale (dBm), which is more relevant for RF performance analysis. Using the formula above, the linear IMD3 values are converted into dBm. The A-SD-HJ-DD-UTFET exhibits more negative IMD3 values compared to the HJ-DD-UTFET, signifying reduced distortion at all gate voltages. This logarithmic representation highlights the superior performance of the asymmetric design, especially at higher gate voltages, where distortion typically becomes more prone in conventional devices.

The Fig. 10b depicts the 1-dB compression point variation with gate voltage. The 1-dB compression point should be high to ensure that the device operates linearly over a broader range of input powers. The 1-dB compression point indicates the input power level at which the device’s gain drops by 1 dB due to nonlinearity. The A-SD-HJ-DD-UTFET achieves higher compression point across the gate voltage range, indicating that it can handle larger input signal power before significant distortion occurs. This result is particularly important in RF applications for maintaining linearity under varying power levels.

## RF, analog and linearity performance of biosensor

Though various FET-based biosensors have been developed for biomolecule and virus detection^84–87^, no reported work has specifically analyzed the RF/analog, and linearity performance of these viral analytes. These performance metrics are critical for biosensing applications, as high RF performance enables rapid signal processing, superior analog characteristics ensuring accurate detection, and excellent linearity. This minimizes distortion for precise biosensing. In FET-based biosensors, biomolecules entering the nanogap alter the local dielectric constant, significantly impacting the device’s electrical characteristics^88^. To mitigate this effect, the proposed A-SD-HJ-DD-UTFET biosensor incorporates an insulating material within the nanogap that aligns with the dielectric constant (κ) of the target biomolecule. For instance, dengue NS1 proteins (κ = 78.7) induce stronger gate-induced electric field modulation compared to SARS-CoV (κ = 2) or Zika virus (κ = 2.9), enhancing sensitivity and optimizing device performance. The simulated dielectric constant models the effective permittivity reflecting biomolecule concentration in the sensing cavity. Higher biomolecule levels, like dengue NS1 (κ ≈ 78.7), raise the local dielectric constant, which is represented by increased κ values in simulation. This method links biomolecular loading to sensor response, with each dielectric constant corresponding to a specific concentration level. This tailored dielectric matching approach ensures high-speed operation and precise biomolecular detection, making it highly suitable for early dengue diagnosis and effective disease management.

**Fig. 11:**
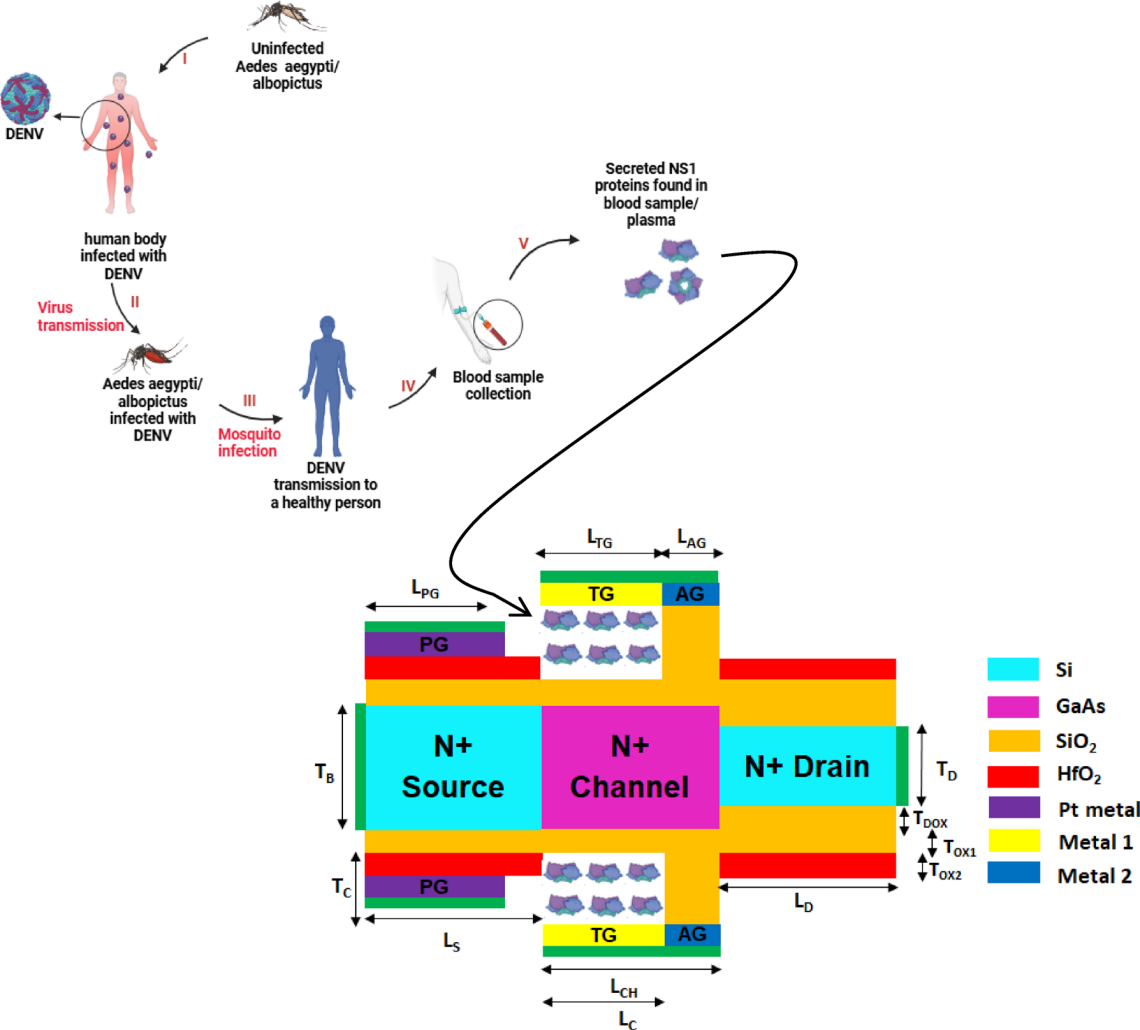
Dengue virus transmission cycle and its integration with the proposed A-SD-HJ-DD-UTFET device as a biosensor.

The nanocavity is strategically placed near the source-channel interface on both top and bottom because band-to-band tunneling (BTBT) in TFETs is highly sensitive to the local gate-channel electrostatics at the source side, which directly modulates the ON OFF current ratio. Introducing the dengue NS1 protein into this cavity modulates the local dielectric environment, thereby enhancing the vertical electric field and improving tunneling probability rather than drain side which offered lower sensitivity. To facilitate this mechanism the HfO_2_ oxide below and above the CG on top and bottom is etched and extended to form an optimized cavity length and thickness 15 nm and 3 nm as given in Fig. 11. These dimensions are sufficient to hold NS1 monomers (≈ 0.51 nm^2^), ensuring biologically relevant embedding. This configuration achieves the maximum I_ON_/I_OFF_ sensitivity of 2.25 × 10^11^ for dengue NS1 protein detection along with experimental biosensor calibration^63^.

**Fig. 12:**
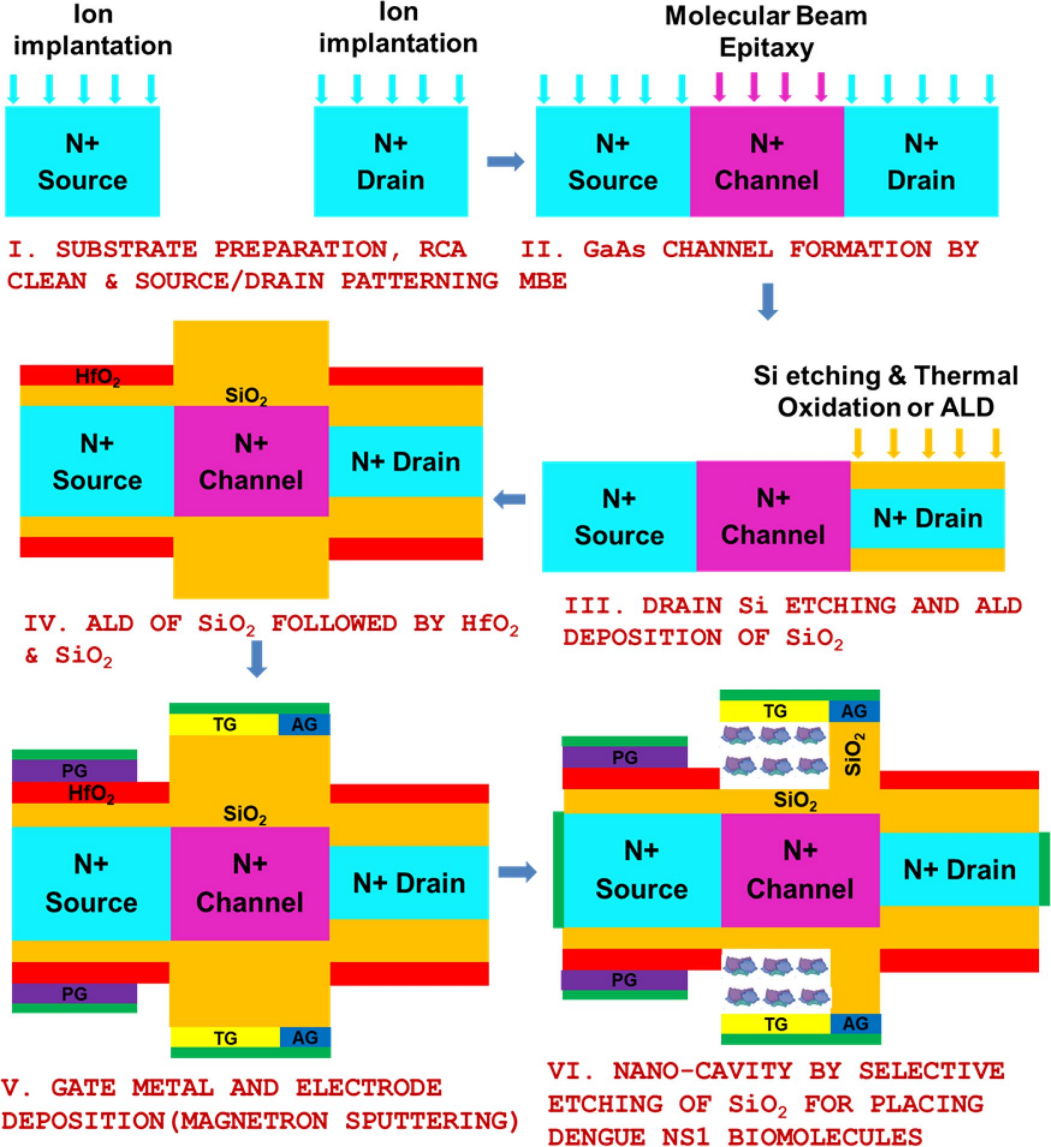
The detailed schematic fabrication process of A-SD-HJ-DD-UTFET biosensor^63^.

**Fig. 13:**
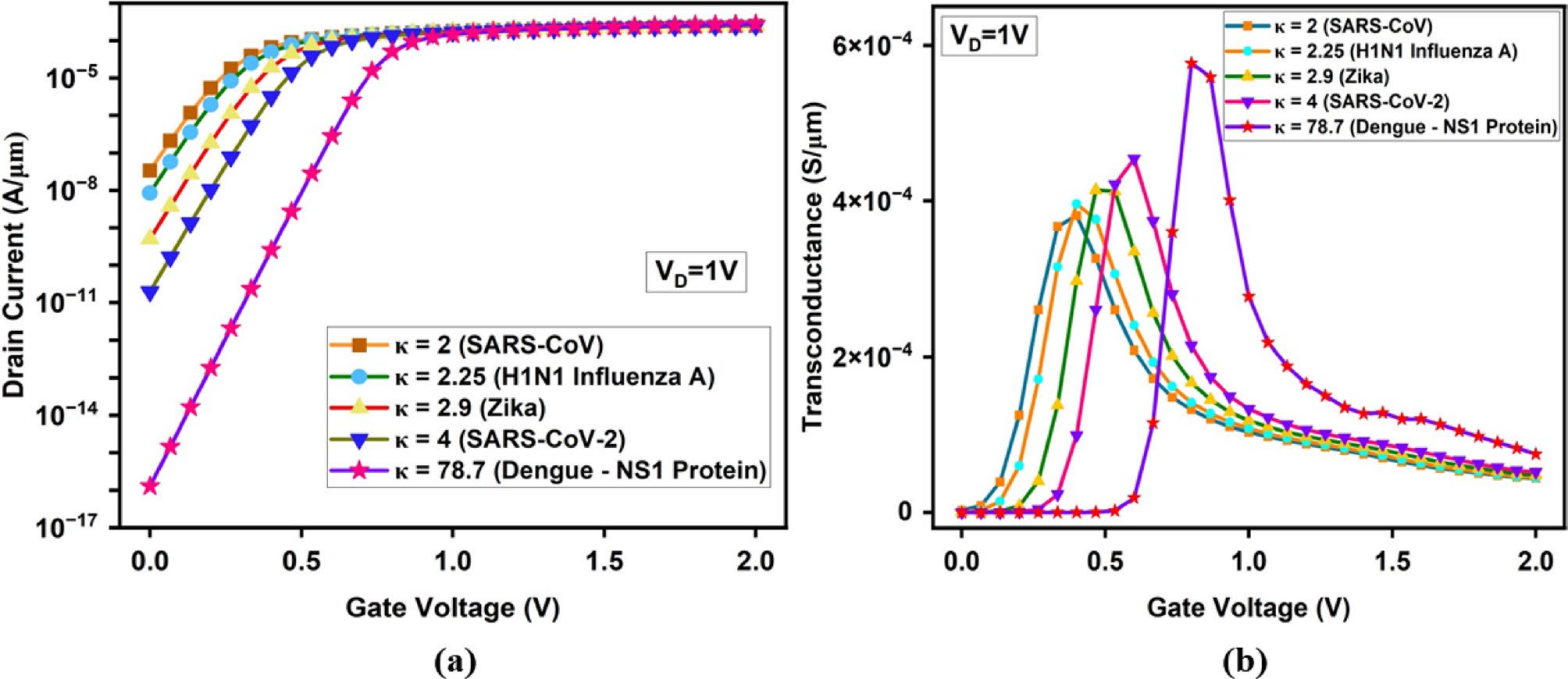
(**a**) I_D_-V_G_ of the proposed A-SD-HJ-DD-UTFET biosensor for various dielectric constants (κ) of biomolecules. (**b**) Transconductance for various κ values of biomolecules.

Also, the dengue virus transmission cycle in Fig. 11 indicates the uninfected Aedes aegypti/albopictus ingesting dengue virus from dengue virus (DENV) infected persons blood as depicted in stage I. It enters the mosquito’s midgut where it infects and multiplies and enters the salivary glands after the incubation period as given in stage II (viral transmission). Then the mosquito spreads DENV into healthy person’s bloodstream through its saliva as given in stage III (mosquito infection). Once the dengue symptoms occur person’s blood sample is collected in stage IV where NS1 proteins will be present as given in stage V which serves as a crucial dengue biomarker for early detection through the proposed A-SD-HJ-DD-UTFET biosensor. The detailed fabrication process with step by step schematic of the A-SD-HJ-DD-UTFET biosensor is given in the below Fig. 12. Figure 13a shows I_D_ – V_G_ comparison for different dielectric constants of biomolecules deadly viruses analytes like SARS CoV Spike glycoprotein, HINI influenza A Hemagglutinin, Zika ssRNA genome, SARS CoV-2 S glycoprotein and dengue NS1 protein whose values are found to be 2^89^, 2.25^90^, 2.9^91^, 4^86^ and 78.7^92,93^.

The RF performance analysis of the A-SD-HJ-DD-UTFET for biosensing applications demonstrates its exceptional capability for detecting dengue NS1 proteins. The transconductance (g_m_) graph exhibits a sharp peak for dengue NS1 proteins, with a maximum value of 5.77 × 10^−4^ S at V_G_ = 0.8 V, compared to 4.54 × 10^−4^ S for SARS-CoV-2 at V_G_ = 0.6 V. The graph shown in Fig. 13b clearly depicts that as V_G_ increases, g_m_ rises sharply to its peak and then decreases due to the saturation of carrier tunneling and the diminishing rate of increase in I_D_. The broader and higher transconductance peak during NS1 detection (577 µS vs. 381 µS for SARS-CoV) highlights enhanced carrier tunneling and gate control in the A-SD-HJ-DD-UTFET biosensor. This improvement, driven by the asymmetric source/drain design and heterojunction integration, boosts transconductance-to-current sensitivity—crucial for biosensing.

**Fig. 14:**
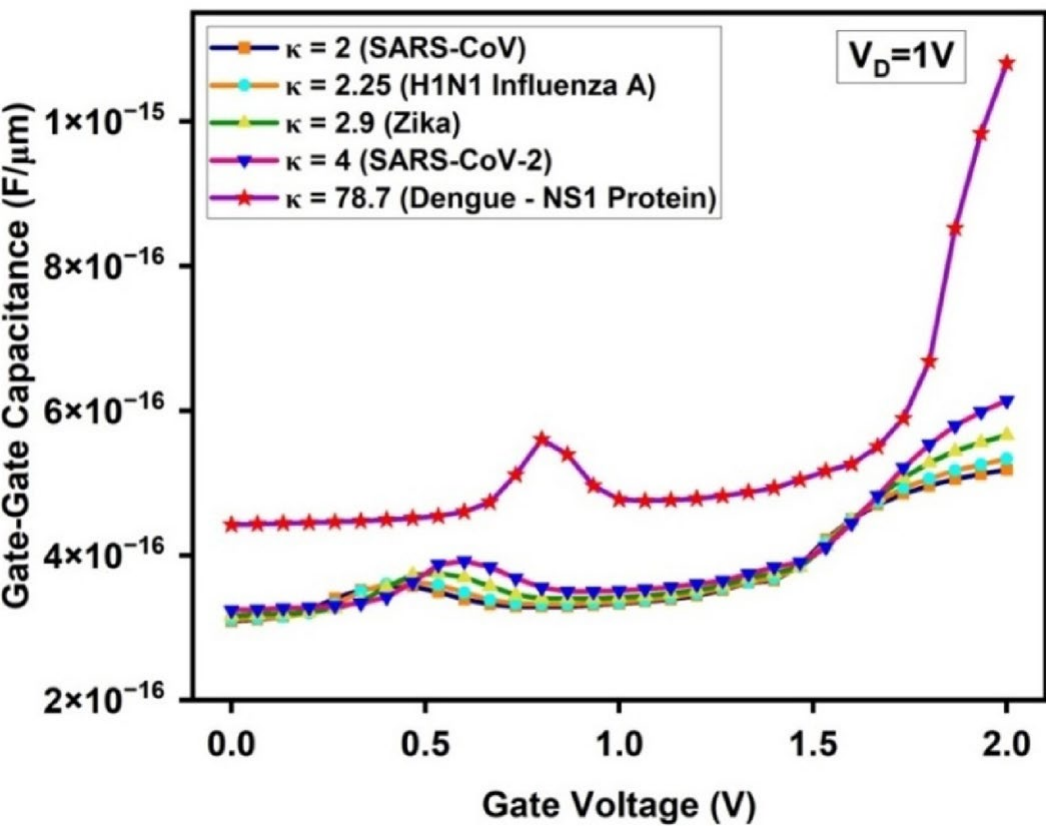
Gate-Gate Capacitance (C_gg_) comparison for various dielectric constants (κ) of biomolecules.

The Gate-Gate Capacitance (C_gg_) graph (Fig. 14) shows a gradual increase with V_G_, followed by a steeper rise for dengue NS1 proteins beyond V_G_ = 1.0 V. The observed peak in the C_gg_ plot particularly for κ = 78.7 (Dengue–NS1 protein), corresponds to the gate voltage region where maximum inversion occurs, resulting in strong accumulation of carriers at the tunneling junction. At this stage, the device experiences enhanced gate-to-channel coupling, leading to an increase in charge storage capability and thus peak gate capacitance (reaching up to 1.08 × 10^–15^ F/µm). This phenomenon is more predominant in the dengue NS1 case due to the high dielectric constant, which amplifies the vertical electric field and boosts carrier confinement near the tunneling region. Beyond this point, the channel reaches electrostatic saturation, causing the C_gg_ to either flatten or increase moderately based on further gate voltage increments.

**Fig. 15:**
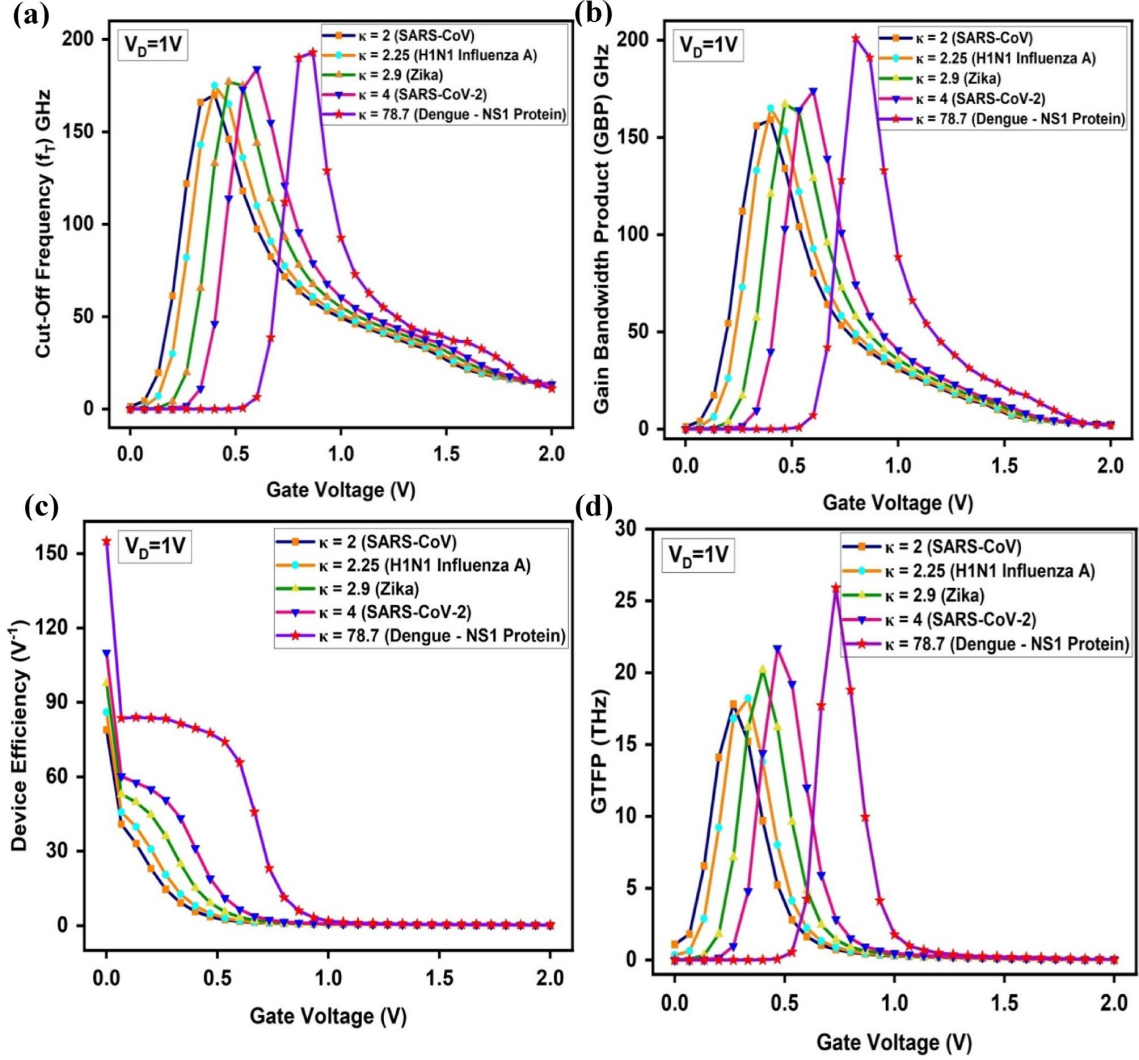
Comparison of (**a**) Cut-Off Frequency (f_T_) (**b**) Gain Bandwidth Product (GBP) (**c**) Device Efficiency (TGF) (**d**) Gain Transconductance Frequency Product (GTFP) for the proposed A-SD-HJ-DD-UTFET biosensor.

The Cut-Off frequency (f_T_) graph exhibits a sharp rise to its peak for all biomolecules, followed by a gradual decline. For dengue NS1 proteins, f_T_ achieves a maximum of 193 GHz at V_G_ = 0.866 V, which is 4.89% higher than the 184 GHz peak observed for SARS-CoV-2 at V_G_ = 0.6 V as given in Fig. 15a. This gate voltage range is driven by the transconductance and gate capacitance saturation. The highly polarized dielectric cavity fully activates the tunneling junction at this point and increases in V_G_ have little effect on f_T_, causing a short f_T_ plateau before dropping off, indicative of strong gate-channel coupling and electrostatic saturation.

The GBP values increase as the dielectric constant (κ) of the biomolecule rises, demonstrating enhanced electrostatic coupling and improved device performance. The lowest GBP of 159 GHz is observed for SARS-CoV with κ = 2, while the highest GBP of 201 GHz is recorded for dengue NS1 protein with κ = 78.7 as given in Fig. 15b. The curve for κ = 78.7 (Dengue–NS1 Protein) exhibits a slight flattening between V_G_ = 0.8–0.9 V. This localized increase in dielectric constant from dengue NS1 biomolecules intensifies the vertical electric field at the source-channel junction, boosting tunneling efficiency and ON current. It also improves electrostatic control, reducing leakage and parasitic capacitances due to the asymmetric UTFET structure. Additionally, the Si-GaAs-Si heterostructure strengthens electric field modulation across the channel, further improving charge transport and boosting GBP for high-frequency biosensing.

The Transconductance Generation Factor (TGF), which measures how efficiently gate voltage variations translate into current, follows a similar upward trend with increasing dielectric constant and is represented in the Fig. 15c. In the subthreshold region (V_G_ = 0 to 0.1 V), high κ biomolecules such as dengue NS1 (κ = 78.7), show dramatic enhancement of the vertical electric field near the source-channel interface, sharply enhancing tunneling probability and device efficiency. However, the gate control becomes stronger and the tunneling window saturates for most biomolecules and shifts to V_G_ of 0.1–1 V in the moderate inversion region, except for dengue NS1 protein leading to larger efficiency due to stronger dielectric coupling. At V_G_ > 1 V (1–2 V) the device enters saturation and over the entire gate voltage range (V_G_ = 1–2 V), the device efficiency ceases to increase for all biomolecules (including dengue NS1). The lowest device efficiency of 78.9 V^-1^ is observed for SARS-CoV, while dengue NS1 protein exhibits the highest TGF of 155 V^-1^.

GTFP, which evaluates overall device performance by integrating gain, transconductance, and frequency response, follows the trends of GBP and TGF. Dengue NS1 detection achieves the highest GTFP of 25.9 THz, significantly outperforming SARS-CoV (17.8 THz) as given in Fig. 15d. The optimized TG and AG lengths (15 nm and 5 nm) plays a crucial role in reducing second-order harmonics and parasitic effects, resulting in higher signal clarity and improved device stability.

In addition to RF metrics like f_T_, GBP, and GTFP, linearity plays a vital role in ensuring distortion-free signal processing especially when biosensor outputs are interfaced with analog front-end circuits in real-world diagnostic platforms. For low-concentration viral targets like dengue NS1, linearity ensures that bio-signals remain interpretable without harmonic interference. The interaction of analytes with the sensing surface modifies the local dielectric environment (κ), impacting gate control. Technically, the high dielectric constant (κ = 78.7) of dengue NS1 protein enhances gate-channel electrostatic coupling by increasing the effective gate capacitance at the sensing surface^94^. This can be understood through the relation:

17 $$C_{OX} = \frac{{\kappa \varepsilon_{0} }}{{t_{ox} }}$$

where C_ox_ is the oxide capacitance per unit area, κ is the dielectric constant of the biomolecular layer (here, NS1), ε_0_ is the vacuum permittivity, and t_ox_ is the oxide thickness. A higher κ directly increases C_ox_, resulting in stronger gate control over the channel potential. This reduces the screening effect and allows more effective modulation of the channel with smaller changes in gate voltage. As a result, transconductance (g_m_ = ∂I_D_/∂V_G_) increases because the drain current responds more sensitively to gate voltage. Second-order and third-order harmonic distortions are reduced due to the more linear gate-to-channel control, especially at small signal levels, improving VIP2 and VIP3 metrics. Additionally, improved IIP3 and IMD3 metrics further demonstrate the sensor’s ability to resist intermodulation distortion and maintain signal fidelity across varying inputs. Though these parameters are conventionally used in RF and analog design, they are crucial here to validate the biosensor’s precision, especially in portable and point-of-care dengue diagnostics. The following section explores how the A-SD-HJ-DD-UTFET achieves superior linearity across various biomolecular detections, with a clear advantage for NS1.

**Fig. 16:**
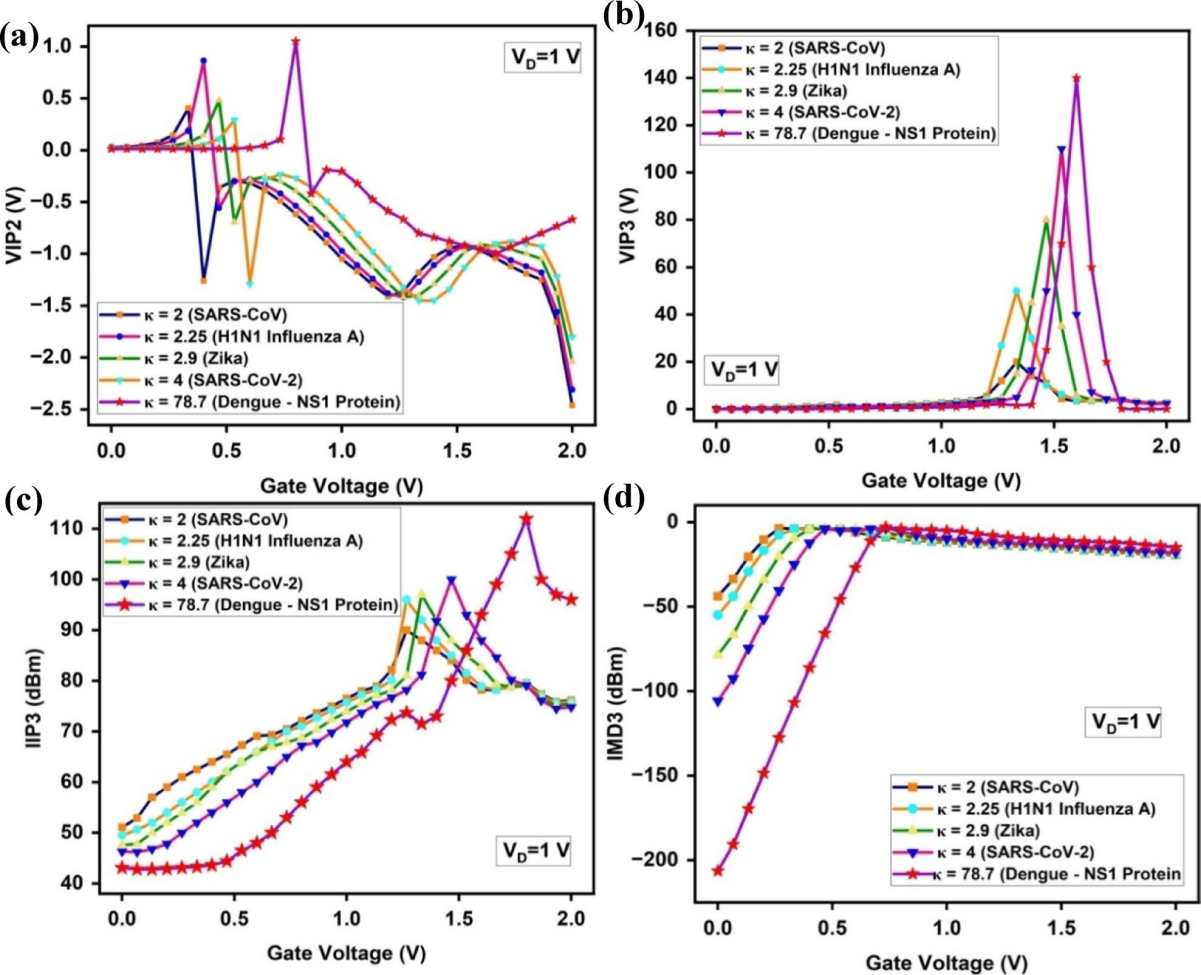
Comparison of (**a**) VIP2 (**b**) VIP3 (**c**) IIP3 (**d**) IMD3 for the proposed A-SD-HJ-DD-UTFET biosensor.

The VIP2 graph in the Fig. 16a showcases the proposed A-SD-HJ-DD-UTFET biosensor capability to suppress second-order distortion for various biomolecules. A higher dip of 1.05 V is observed for dengue NS1 protein at the threshold voltage, indicating minimized second-order nonlinearity. The high dielectric constant (κ = 78.7) of NS1 proteins enhances gate modulation, resulting in superior second-order harmonic suppression. In comparison, biomolecules such as SARS-CoV (κ = 2) and H1N1 Influenza A (κ = 2.25) exhibit much lower VIP2 profiles, reflecting their reduced sensitivity to second-order distortion suppression. The sharper response observed for dengue NS1 proteins highlights the device’s potential for biosensing applications that demand high linearity and precision.

The VIP3 graph, representing the suppression of third-order distortion, exhibits a pronounced peak for dengue NS1 proteins, reaching nearly 140 V at V_G_ = 1.5 V as observed in Fig. 16b. This value is significantly higher than those for SARS-CoV-2 (κ = 4) and Zika virus (κ = 2.9). The high VIP3 values for NS1 proteins can be attributed to the enhanced gate modulation achieved through the device’s asymmetrical design, which includes variations in TG and AG lengths, as well as reduced drain thickness. The broader and higher peak for dengue NS1 proteins ensures lower distortion over a wider range of gate voltages, reinforcing the device’s effectiveness in biosensing applications.

he IIP3 (dBm) graph in Fig. 16c provides insights into the input linearity performance of the device. For dengue NS1 protein, IIP3 (dBm) achieves a peak of approximately 112 dBm at V_G_ = 1.4 V, significantly surpassing the values for other biomolecules. The high dielectric constant of NS1 proteins contributes to enhanced electric field modulation, thereby improving input linearity and ensuring efficient carrier transport. This makes the A-SD-HJ-DD-UTFET highly suitable for applications that require both high sensitivity and high linearity, particularly in detecting dengue NS1 proteins.

In biosensors, lower IMD3 ensures that the detected signal remains free from unwanted distortions, leading to higher accuracy in biomolecule identification. In the proposed A-SD-HJ-DD-UTFET biosensor a lowest IMD3 value of -206 dBm is observed for dengue NS1 protein, while SARS-CoV exhibits the highest distortion at -43.8 dBm as represented in Fig. 16d. This substantial improvement for NS1 detection is mainly due to its high dielectric constant of the NS1 protein (κ = 78.7), which strengthens electrostatic coupling and enhances charge modulation. As a result, carrier transport becomes more stable, reducing signal distortion and improving overall linearity.

Also, a crucial higher 1 dB compression point in biosensors allows accurate biomolecular detection even at higher signal strengths, preventing saturation-related errors**.** A higher 1 dB compression point signifies better power handling and reduced signal degradation.

**Fig. 17:**
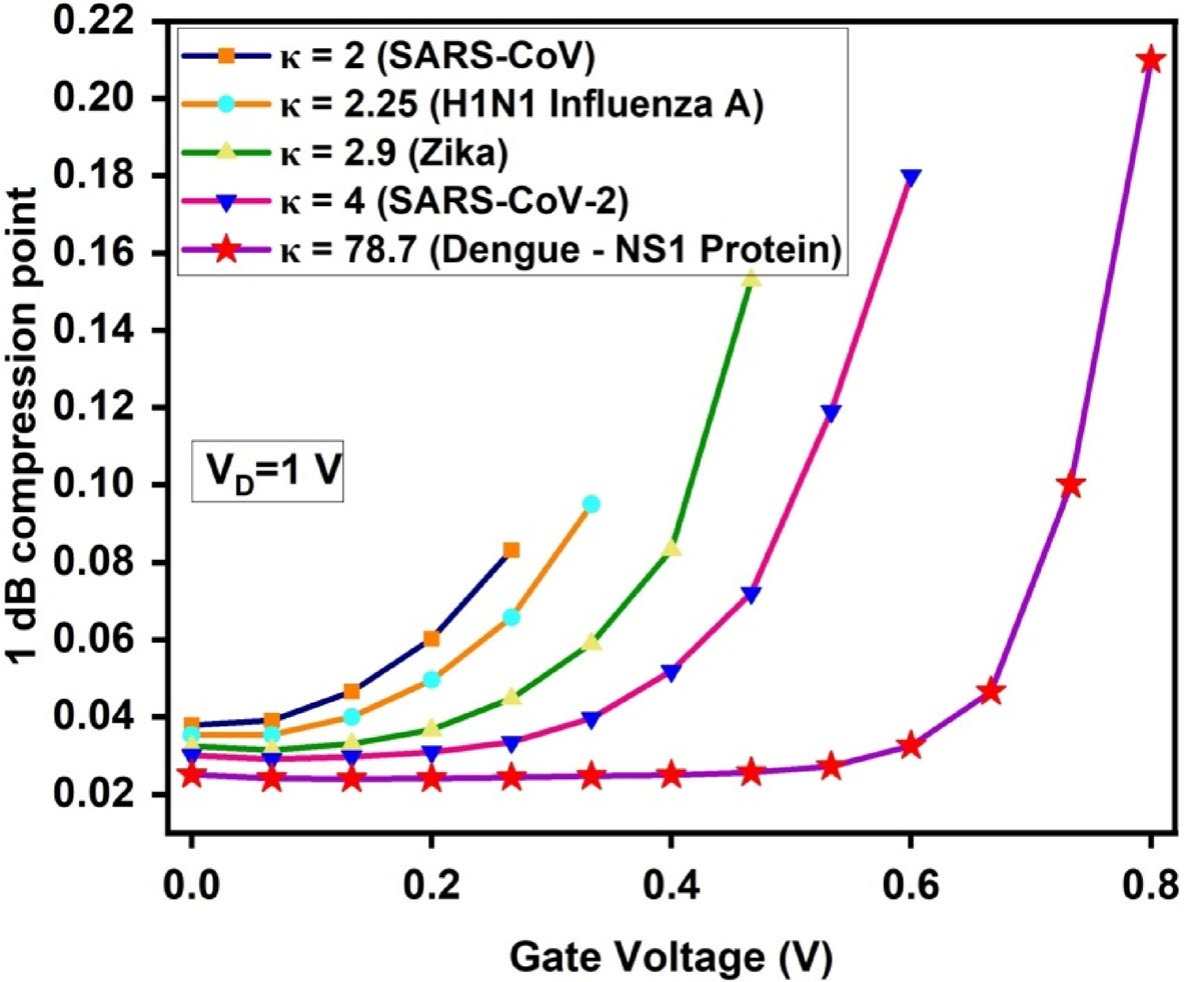
1-dB Compression point of the proposed A-SD-HJ-DD-UTFET biosensor.

As observed in Fig. 17, dengue NS1 protein achieves the highest 1 dB compression point of 0.21 V, while SARS-CoV records the lowest at 0.083 V. The higher dielectric constant of NS1 improves surface potential modulation and transconductance, allowing the device to maintain linear amplification over a broader power range, reducing gain compression effects.

The overall analog/RF parameters of the proposed A-SD-HJ-DD-UTFET biosensor are tabulated in the below Table 4. Table 4 implies that the proposed A-SD-HJ-DD-UTFET biosensor offers enhanced analog/RF and linearity performance for dengue NS1 protein than the other viruses. Also Table 5 presents a comparative overview of TFET-based biosensors employing dielectric modulation for biomolecular detection, demonstrating that the proposed A-SD-HJ-DD-UTFET biosensor outperforms existing designs with the highest ON/OFF current ratio (2.83 × 10^12^), validating its superior sensitivity for dengue NS1 protein detection surpassing conventional biosensors^95–99^.

**Table 4:** Comparison of various analog/RF Figure of Merits of various dielectric constants of biomolecules for the proposed A-SD-HJ-DD-UTFET biosensor.

Virus name	Analytes used	Dielectric constant (κ)	g_m_ (µS)	f_T_ (GHz)	GBP (GHz)	TGF (V^-1^)	GTFP (THz)
SARS-CoV	Spike glycoprotein	2	381	170	159	78.9	17.8
H1N1 Influenza A	Hemagglutinin	2.25	396	175	165	86	18.2
Zika Virus	SsRNA genome	2.9	414	177	167	97.8	20.2
SARS-CoV-2	S glycoprotein	4	454	184	174	110	21.7
Dengue virus	NS1 protein	78.7	577	193	201	155	25.9

**Table 5:** Comparison of TFET-based biosensors employing dielectric modulation for protein or biomolecule detection.

Name of the biosensor	V_G_,V_D_ (V) and κ value	Name of the analyte	Gate length (nm)	ON current (A/µm)	OFF current (A/µm)	I_ON_/I_OFF_ ratio	Refs.
Si/InAs/Ge JLTFET biosensor	0.5, 0.5 & 12	Dielectrics	20	1.15 × 10^–5^	1.12 × 10^–14^	1.75 × 10^9^	^77^
DMG-HJ-SOI-TFET biosensor	0.5, 0.5 & 12	CREB2 protein	15	~ 10^–5^	10^–15^	10^10^	^100^
FFC Bio-TFET biosensor	1, 1 & 12	Gelatin	50	~ 10^–8^	10^–11^	10^3^	^101^
DG JLTFET biosensor	1, 1 & 7	Outer shell of human protein	20	~ 10^–7^	5 × 10^–15^	10^8^	^85^
Poc-MGOT-FET	1.5, 1 & 32	T47D cancer cell	50	10^–5^	~ 10^–16^	10^11^	^95^
DG-bioHTFET	1.5, 1 & 5	Low hydrated SARS CoV-2 protein	20	2.32 × 10^–5^	~ 10^–9^	3.55 × 10^5^	^102^
A-SD-HJ-DD-UTFET biosensor	2, 1 & 78.7	Dengue NS1 protein	20	2.3 × 10^–4^	8.12 × 10^–17^	2.83 × 10^12^	This work

JLTFET - Junctionless TFET, DMG-HJ-SOI-TFET - Dual Material Gate InSb/Si (Indium Antimonide/silicon) Heterojunction Silicon on Insulator TFET, FFC Bio-TFET - Fringe Field Capacitance Bio-TFET, DG JLTFET - Double Gate JLTFET, Poc-MGOTFET - Pocket Modified Gate Oxide TFET, DG-bioHTFET - Dielectrically Modulated Double Gate Bio-Heterojunction TFET.

## Advancing RF biosensing: high-frequency detection with TCAD optimization

Conventional RF biosensors like Split Ring Resonator (SRR)-based, Interdigitated Capacitor (IDC)-based, Nuclear Magnetic Resonance (NMR)-RFIC, and PCB-resonator-based sensors make use of passive resonance tuning or impedance changes in detecting biomolecules. Most RF biosensors proposed to date operate at relatively low frequencies, typically around 2 GHz or lower, such as those for glucose, heparin, IgG, melanoma, and cardiopulmonary monitoring^103–107^. However, such low-frequency operation limits their suitability for Point-Of-Care Testing (POCT) because it results in lower sensitivity, larger sensor sizes, and reduced resolution for biomolecular interactions^108^. Higher frequencies enable better miniaturization, enhanced sensitivity, and improved rapid detection capabilities, making them more suitable for POCT applications.

Only a few commercially available RF biosensors exist, such as Glucowise, a non-invasive glucose monitoring device, and Biacore systems, which use surface plasmon resonance (SPR) for biomolecular detection^109^. These commercially available RF biosensors are focused on glucose sensing or general biomolecular interactions, with limited developments in infectious disease detection.

The proposed A-SD-HJ-DD-UTFET-based dengue NS1 biosensor, whose cut-off frequency is 193 GHz, surpasses the current shortcomings with high transconductance, ultra-low OFF-state leakage, and an asymmetric structure that improves RF performance. Its RF parameters have been exhaustively analyzed with the aid of TCAD simulations, which provide a device-level analysis capable of giving greater insights into charge transport, carrier behaviour, transconductance fluctuations, surface potential mapping, and noise modeling.

In contrast to traditional S-parameter analysis, which mainly analyzes network response (reflection, transmission, and impedance matching) and necessitates advanced equipment like a Vector Network Analyzer (VNA) and Semiconductor Parameter Analyzer (SPA), TCAD-based RF analysis facilitates accurate, affordable, and scalable evaluation at the initial stage before prototyping and fabrication. It permits optimization of electrical properties at the atomic level with great precision, maximizing biosensor sensitivity and response time. By taking advantage of high-frequency RF detection and TCAD-driven performance analysis, the developed biosensor provides a new standard for real-time, ultra-sensitive, and label-free dengue diagnosis.

Table 6 shows a comparative overview of existing POCT biosensors and commercial diagnostic kits for DENV NS1 detection^110–113^. The proposed A-SD-HJ-DD-UTFET biosensor demonstrates superior sensitivity, faster response, lower cost, and serotype-independent detection, highlighting its potential as an effective alternative for next-generation dengue diagnostics.

**Table 6:** Benchmarking of the proposed A-SD-HJ-DD-UTFET biosensor against existing POCT biosensing technologies and commercial diagnostic kits for DENV NS1 detection.

Various POCT biosensors/commercial diagnostic tests	Analyte	Sensing method	Advantages	Disadvantages	Refs.
Electrochemical biosensor	Nucleotide sequence	Electrochemical sensing	Miniaturization and Potential multiplexing	Requires complex surface functionalization and target amplification	^114^
Lateral flow strips	DENV NS1	Immunochromato-graphic Test (ICT)	Portable and User friendly	Lower sensitivity for certain DENV serotypes	^115,116^
Microfluidic Chips	DENV serum & NS1	Lab-on-chip with microchannels	Compact and low sample requirement	Fabrication complexities	^117,118^
Optical genosensor	RNA-DNA	Optical (SPR or Fluorescence)	Effective for DNA-RNA hybridization	Requires a longer time and Expensive instrumentation	^119^
PanBio dengue early ELISA Kit	DENV NS1	Enzyme-Linked Immunosorbent Assay	Provides relatively rapid results (~ 2 to 3 h)	Lower Sensitivity, particularly during very early infection	^120^
Dengue NS1 Ag Strip kit	DENV NS1	ICT Technique	Higher specificity than some ELISA kits	Lower Sensitivity compared to standard ELISA techniques	^121^
Omega capture ELISA (IgM assay)	IgM	Immobilized anti-IgM antibody on ELISA plate	Higher specificity for recent infections	Lower sensitivity, Requires Febrile samples (above 5 days)	^122^
SD Bioline IgG	IgG	Immobilized anti-IgG antibody on lateral flow strip	Moderate sensitivity and specificity, Easy to operate	High cost and not suitable for early detection	^123^
FET Based Biosensor (A-SD-HJ-DD-UTFET)	DENV NS1	Dielectric modulation based sensing	Higher sensitivity, Early detection, Low cost, Rapid response and Serotype & Label-free detection	Yet to be commercialized	This work

## Conclusions

With biosensing revolutionizing implications, this research presents a first-of-its-kind investigation of symmetric and asymmetric Source-Drain Heterojunction Dual-Dielectric Uniform Tunnel FETs (A-SD-HJ-DD-UTFET) for analog, radio frequency, and linearity applications. The asymmetric device structure provides unmatched electrostatic control with enhanced tunnelling efficiency and OFF-state leakage current suppression. It is characterized by well-designed longer TG and shorter AG lengths along with a thinner drain. The integration of a Si-GaAs heterojunction at the source-drain interface further refines carrier transport dynamics, significantly improving the I_ON_/I_OFF_ ratio, transconductance, and overall RF performance, solidifying its suitability for ultra-high frequency, low power applications. Beyond the RF innovations, the A-SD-HJ-DD-UTFET emerges as an advancement in biosensing, particularly for the early detection of dengue NS1 protein, a crucial biomarker for timely intervention. This biosensor exhibits an exceptional RF performance such as g_m_ of 577 µS, a f_T_ of 193 GHz, GBP of 201 GHz, far surpassing conventional biosensors. With an outstanding device efficiency of 155 V⁻^1^ and GTFP reaching up to 25.9 THz, the proposed UTFET based biosensor sets a new benchmark for high-sensitivity, real-time, and label-free biomolecular detection. This aligns with the growing demand for rapid, ultra-sensitive detection of biomolecular interactions, paving the way for semiconductor-driven biosensor revolutionization. By harnessing the unique advantages of asymmetric UTFETs, this study spearheads the next generation of biosensing platforms, seamlessly integrating semiconductor innovation with critical healthcare applications.

## Data Availability

No/Not applicable (this manuscript does not report data generation or analysis). For any data-related inquiries, please contact the corresponding author. Third party material—No, all the material is owned by the authors and/or no permissions are required.
